# Heterogeneous nuclear ribonucleoprotein K promotes cap-independent translation initiation of retroviral mRNAs

**DOI:** 10.1093/nar/gkad1221

**Published:** 2024-01-02

**Authors:** Yazmín Fuentes, Valeria Olguín, Brenda López-Ulloa, Dafne Mendonça, Hade Ramos, Ana Luiza Abdalla, Gabriel Guajardo-Contreras, Meijuan Niu, Barbara Rojas-Araya, Andrew J Mouland, Marcelo López-Lastra

**Affiliations:** Laboratorio de Virología Molecular, Instituto Milenio de Inmunología e Inmunoterapia, Departamento de Enfermedades Infecciosas e Inmunología Pediátrica, Escuela de Medicina, Pontificia Universidad Católica de Chile, Marcoleta 391, Santiago, Chile; Laboratorio de Virología Molecular, Instituto Milenio de Inmunología e Inmunoterapia, Departamento de Enfermedades Infecciosas e Inmunología Pediátrica, Escuela de Medicina, Pontificia Universidad Católica de Chile, Marcoleta 391, Santiago, Chile; Laboratorio de Virología Molecular, Instituto Milenio de Inmunología e Inmunoterapia, Departamento de Enfermedades Infecciosas e Inmunología Pediátrica, Escuela de Medicina, Pontificia Universidad Católica de Chile, Marcoleta 391, Santiago, Chile; Laboratorio de Virología Molecular, Instituto Milenio de Inmunología e Inmunoterapia, Departamento de Enfermedades Infecciosas e Inmunología Pediátrica, Escuela de Medicina, Pontificia Universidad Católica de Chile, Marcoleta 391, Santiago, Chile; Laboratorio de Virología Molecular, Instituto Milenio de Inmunología e Inmunoterapia, Departamento de Enfermedades Infecciosas e Inmunología Pediátrica, Escuela de Medicina, Pontificia Universidad Católica de Chile, Marcoleta 391, Santiago, Chile; HIV-1 RNA Trafficking Laboratory, Lady Davis Institute at the Jewish General Hospital, Montréal, Quebec H3T 1E2, Canada; Department of Microbiology and Immunology, McGill University, Montreal, Quebec H4A 3J1, Canada; HIV-1 RNA Trafficking Laboratory, Lady Davis Institute at the Jewish General Hospital, Montréal, Quebec H3T 1E2, Canada; Department of Medicine, McGill University, Montreal, Quebec H4A 3J1, Canada; HIV-1 RNA Trafficking Laboratory, Lady Davis Institute at the Jewish General Hospital, Montréal, Quebec H3T 1E2, Canada; Laboratorio de Virología Molecular, Instituto Milenio de Inmunología e Inmunoterapia, Departamento de Enfermedades Infecciosas e Inmunología Pediátrica, Escuela de Medicina, Pontificia Universidad Católica de Chile, Marcoleta 391, Santiago, Chile; HIV-1 RNA Trafficking Laboratory, Lady Davis Institute at the Jewish General Hospital, Montréal, Quebec H3T 1E2, Canada; Department of Microbiology and Immunology, McGill University, Montreal, Quebec H4A 3J1, Canada; Department of Medicine, McGill University, Montreal, Quebec H4A 3J1, Canada; Laboratorio de Virología Molecular, Instituto Milenio de Inmunología e Inmunoterapia, Departamento de Enfermedades Infecciosas e Inmunología Pediátrica, Escuela de Medicina, Pontificia Universidad Católica de Chile, Marcoleta 391, Santiago, Chile

## Abstract

Translation initiation of the human immunodeficiency virus-type 1 (HIV-1) genomic mRNA (vRNA) is cap-dependent or mediated by an internal ribosome entry site (IRES). The HIV-1 IRES requires IRES-transacting factors (ITAFs) for function. In this study, we evaluated the role of the heterogeneous nuclear ribonucleoprotein K (hnRNPK) as a potential ITAF for the HIV-1 IRES. In HIV-1-expressing cells, the depletion of hnRNPK reduced HIV-1 vRNA translation. Furthermore, both the depletion and overexpression of hnRNPK modulated HIV-1 IRES activity. Phosphorylations and protein arginine methyltransferase 1 (PRMT1)-induced asymmetrical dimethylation (aDMA) of hnRNPK strongly impacted the protein's ability to promote the activity of the HIV-1 IRES. We also show that hnRNPK acts as an ITAF for the human T cell lymphotropic virus-type 1 (HTLV-1) IRES, present in the 5′UTR of the viral sense mRNA, but not for the IRES present in the antisense spliced transcript encoding the HTLV-1 basic leucine zipper protein (sHBZ). This study provides evidence for a novel role of the host hnRNPK as an ITAF that stimulates IRES-mediated translation initiation for the retroviruses HIV-1 and HTLV-1.

## Graphical Abstract

**Graphical Abstract:**
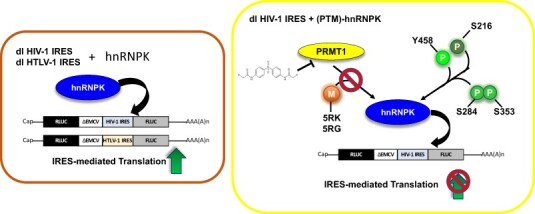

## Introduction

The human immunodeficiency virus-type 1 (HIV-1), the causative agent of the acquired immune deficiency syndrome (AIDS) pandemic, elicits multiple strategies to guarantee the initiation of viral mRNA (vRNA) translation (1). These strategies ensure the synthesis of the structural polyproteins Gag and Gag/Pol, required for progeny virus production. Translation initiation of the HIV-1 vRNA occurs via canonical and non-canonical cap-dependent mechanisms or by using an internal ribosome entry site (IRES)-dependent mechanism (1–3). The HIV-1 vRNA harbors two IRESs, the HIV-1 IRES (the main focus of this study) within the 5′untranslated region (UTR) and the Gag-IRES within the *gag*-coding region (4–6).

In cells, translational control is primarily exerted during the initiation step of protein synthesis, a multistep process that leads to the assembly of the 80S ribosome at the start codon of the mRNA (7). The first step in canonical translation initiation involves 5′cap (m7GpppN) recognition by the eukaryotic initiation factor (eIF)4F, a heterotrimeric complex comprising the cap-binding protein, eIF4E, an ATP-dependent RNA helicase, eIF4A, and the scaffold protein, eIF4G (7). Also, eIF4G bridges the interaction with the 40S ribosomal subunit via eIF3 and interacts with the poly(A)-binding protein (PABP) that covers the poly(A) tail, stimulating translation initiation, translation reinitiation through ribosome recycling and enhancing mRNA stability. HIV-1 targets cap-dependent translation initiation during replication (1,2) such that in HIV-1 replicating cells, eIF4E and the eIF4E-binding protein are hypophosphorylated, reducing cap-dependent translation initiation (8). Also, the HIV-1 protease cleaves eIF4G and PABP, impeding eIF4E-eIF4G and eIF4G-PABP interactions, negatively impacting cap-dependent translation initiation (9). Furthermore, HIV-1 replication induces oxidative and osmotic stress and blocks the cell cycle in G2/M, physiological conditions in which cap-dependent translation initiation is hindered (8,10–12). While HIV-1 inhibits cap-dependent translation initiation, non-canonical translation initiation sustains Gag polyprotein synthesis (4,13,14). The HIV-1 IRES remains functional when cap-dependent translation initiation is repressed by drug-induced cell cycle blockage in G2/M (4), by the expression of the poliovirus (PV) 2A or the foot and mouth disease virus (FMDV) L proteases, viral enzymes that cleave eIF4G, specifically inhibiting cap-dependent translation (4,13,14), and in cells where HIV-1 and poliovirus co-replicate enabling HIV-1 Gag protein synthesis (14).

The molecular mechanisms driving HIV-1 IRES activity are still poorly understood (1,2). However, function of the HIV-1 IRES relies on host proteins such as eIF4A and eIF5A (15–17), the ribosomal protein S25 (18), and on IRES *trans-acting factors* (ITAFs) that activate or repress its activity (6,11,15,19–23). The known ITAFs for the HIV-1 IRES include the heterogeneous nuclear ribonucleoprotein (hnRNP) A1 (11,21), Staufen1 (22), the human Rev-interacting protein (hRIP) (15), DDX3 (15), the Human antigen R (HuR) (19), and upstream of N-ras (unr) (23). Pulldown/mass spectrometry experiments have identified additional proteins that bind the 5′UTR of the HIV-1 vRNA (24). Among the identified proteins, several members of the hnRNP family of RNA-binding proteins (RBP), including hnRNPA1, hnRNPI, hnRNPK, hnRNPU, and hnRNPF, are found (24). However, except for hnRNPA1 and hnRNPI (11,21,25), the roles of other hnRNPs identified as binding partners of the HIV-1 5′UTR in HIV-1 IRES-mediated translation initiation have not been evaluated. From the hnRNPs identified to bind the HIV-1 vRNA 5′UTR (24), we were interested in assessing the role of the ubiquitously expressed, multifunctional nucleocytoplasmic shuttling RBP, hnRNPK, on HIV-1 IRES activity because of its many roles in HIV-1 gene expression (26–29), but heretofore unresolved function in IRES-mediated vRNA translation initiation. Consistent with this role in viral gene expression, the knockdown of hnRNPK in cells actively replicating HIV-1 decreases intracellular viral proteins and reduces HIV-1 production (28). Other factors that pointed to hnRNPK as an interesting target for this study was that the protein is an ITAF for other viral and cellular IRESs (30–32), and in cells, it forms protein-protein complexes with known HIV-1 IRES ITAFs such as hnRNPA1, HuR, and DDX3, and interacts with other proteins required for HIV-1 IRES function, such as eS25 (33,34).

This study reveals that hnRNPK promotes HIV-1 IRES activity. Our results show that post-translational modifications (PTMs) of hnRNPK modulate the protein's ability to stimulate HIV-1 IRES-mediated translation initiation. Our findings demonstrate that hnRNPK also promotes the activity of the IRESs present within the 5′UTR of human T cell lymphotropic virus-type 1 (HTLV-1) vRNA, without having any impact on the activity of the IRES present in the HTLV-1 antisense RNA, encoding for the HTLV-1 basic leucine zipper (HBZ) protein.

## Materials and methods

### Plasmids

The pNL4.3 DNA (HIV-1 vector; GenBank: AF324493) was obtained through the NIH AIDS Reagent Program, Division of AIDS, NIAID, NIH. The HIV-1 pNL-4.3-RLuc provirus and the plasmid pEGFP-C1 were kindly provided by Dr. R. Soto-Rifo (Laboratorio de Virología Molecular y Celular, Programa de Virología, Instituto de Ciencias Biomédicas, Universidad de Chile, Santiago, Chile) and were described in detail (35). The dual-luciferase (dl) plasmids dl HIV-1 IRES, harboring the 5′UTR of the HIV-1 vRNA (1-336), dl HIV-1 IRES 104–336, dl HIV-1 IRES 1–104, ΔSV40 dl HIV-1 IRES, dl HTLV-1 IRES, and dl sHBZ IRES were described (4,36–38). The hnRNPK plasmid (pCMV-HA-hnRNPK) was purchased from Sino Biological Inc. (#HG16029-NY, Wayne, PA, USA). The selected hnRNPK substitutions have been previously described (39–44). Mutants were generated using the Thermo Fisher Scientific Phusion Site-Directed Mutagenesis Kit (#F-541, Thermo Fisher Scientific Inc. Life Technologies Inc., Carlsbad, CA, USA) using primers described in Table 1. The polymerase chain reaction (PCR) assays were performed in a Veriti TM 96-well Thermal Cycler (#4375768, Thermo Fisher Scientific Inc.). All constructs used in this study were verified by sequencing (Psomagen Inc., Rockville, MD, USA). The hnRNPK plasmids, GFP-hnRNPK and GFP-hnRNPK-5RG, were generously provided by Dr. A. Ostareck-Lederer (Department of Intensive Care Medicine, University Hospital RWTH Aachen, Aachen, Germany) and have been described (44).

**Table 1.** Primers used to generate the hnRNP K point mutants

Mutant	Primers sequences
HNRNP K S284A	Fw: 5′(P)TGATGATATGGCCCCTCGTCGA
	Rv: 5′(P)TAATCTCTTCTAGATGGAGGCATGGG
HNRNP K S284D	Fw: 5′(P)TGATGATATGGACCCTCGTCGA
	Rv: 5′(P)TAATCTCTTCTAGATGGAGGCATGGG
HNRNP K S353A	Fw: 5′(P)AGATACATGGGCCCCATCAGAA
	Rv: 5′(P)ATTGCAGAGTCCCAAGTTTCATC
HNRNP K S353D	Fw: 5′(P)AGATACATGGGACCCATCAGAA
	Rv: 5′(P)ATTGCAGAGTCCCAAGTTTCATC
HNRNP K S216A	Fw: 5′(P)CTTATATCTGAGGCTCCCAT CAAAGGACGT
	Rv: 5′(P)ATCAAGGATGATCTTTATGCA CTCTACAAC
HNRNP K S216D	Fw: 5′(P)CTTATATCTGAGGATCCCATC AAAGGACGT
	Rv: 5′(P)ATCAAGGATGATCTTTATGCA CTCTACAAC
HNRNP K Y458A	Fw: 5′(P)AGTGTGAAGCAGGCTGCA GATGTTGAA
	Rv: 5′(P)GTTCTGCAGCAAATACTGTGCATTCTGTATCTG
HNRNP K Y458D	Fw: 5′(P)AGTGTGAAGCAGGATGCAGATGTTGAA
	Rv: 5′(P)GTTCTGCAGCAAATACTGTGCATTCTGTATCTG
HNRNP K Y458F	Fw: 5′(P)AGTGTGAAGCAGTTTGCAGATGTTGAA
	Rv: 5′(P)GTTCTGCAGCAAATACTGTGCATTCTGTATCTG
HNRNP K Y458T	Fw: 5′(P)AGTGTGAAGCAGACTGCAGATGTTGAA
	Rv: 5′(P)GTTCTGCAGCAAATACTGTGCATTCTGTATCTG
HNRNP K Y458S	Fw: 5′(P)AGTGTGAAGCAGAGTGCAGATGTTGAA
	Rv: 5′(P)GTTCTGCAGCAAATACTGTGCATTCTGTATCTG
HNRNP K Y458E	Fw: 5′(P)AGTGTGAAGCAGGAGGC AGATGTTGAA
	Rv: 5′(P)GTTCTGCAGCAAATACTGTGCATTCTGTATCTG
HNRNP K Y458R	Fw: 5′(P)AGTGTGAAGCAGAGAGCAGATGTTGAA
	Rv: 5′(P)GTTCTGCAGCAAATACTGTGCATTCTGTATCTG
HNRNP K R256K	Fw: 5′(P)TTTCCCATGAAGGGAAGAGGTGGT
	Rv: 5′(P)TCCCACTGGGCGTCCGCG
HNRNP K R299K	Fw: 5′(P)GAGGCGGCAAGGGTGGTAGC
	Rv: 5′(P)GTCCGGGAGGAGGGGGAGG

### Cell culture and drug treatments

HeLa cells (ATCC CCL-2) and human embryonic kidney cells (HEK293T, ATCC, CRL-11268) were grown as previously described in detail (21,22). The PRMT1 inhibitor TC-E 5003 (CAS 17328-16-4, Santa Cruz Biotechnology, Dallas, TX, USA) was diluted in DMSO. HEK293T were seeded at 1.2 × 10^5^ and 6 × 10^4^ per well in a 24- or 48-well culture plates, respectively. 24 h later, cells at 60% confluence were treated with DMSO (control) or TC-E 5003 at the indicated compound concentrations for 24 h.

#### DNA transfection

HEK293T cells were seeded at 1.2 × 10^5^ or 6 × 10^4^ per well in a 24- or 48-well culture plates, respectively. DNA transfection experiments were performed at 70–80% confluency using polyethyleneimine (PEI; GIBCO, Thermo Fisher Scientific Inc.). The cells were cotransfected with 200 ng of dl-plasmids, together with the indicated amount (ng) of HA-hnRNPK plasmids, GFP-hnRNPK plasmids or plasmid pSP64 Poly(A) (#P1241, Promega Corporation, Madison, WI, USA); the last was used as a filler DNA to keep the final DNA concentration constant in all transfection assays. In all experiments, 24 or 48 h post-transfection (hpt), the culture medium was removed, and cells were harvested using Passive Lysis buffer supplied with the Dual-Luciferase® Reporter Assay System (#E1960, Promega Corporation) according to the manufacturer's protocols.

#### siRNA-DNA co-transfection

HEK293T cells were seeded at 1.2 × 10^5^ cells per well in 24-well culture plates. Endogenous hnRNPK protein silencing was performed over 70% confluent cells using the Lipofectamine 2000 system (#11668019, Invitrogen, Thermo Fisher Scientific Inc.). For hnRNPK silencing, a commercially available mix of silencing RNAs that target the hnRNPK open reading frame (ORF), siRNAK (#sc-38282, Santa Cruz Biotechnology), a combination of five target-specific siRNAs against the hnRNPK ORF, or a Dicer-Substrate siRNA (DsiRNA) targeting the 3′UTR of the hnRNPK encoding mRNA (hs.Ri.HNRNPK.13.3, Integrated DNA Technologies, IDT, Coralville, IA, USA), DsiRNAK, were used. As negative controls, a scrambled siRNA (scRNA; #4390844; Ambion, ThermoFisher Scientific Inc) or scDsiRNA (#51-01-14-04, IDT). For RLuc silencing, 50 nM of a duplex siRNA targeting the RLuc open reading frame (siRLuc, 5′-UAUAAGAACCAUUACCAGAUUUGCCUG-3′, (IDT)) was used as described previously (18,21,22,38).

#### Luciferase assays

The activities of firefly luciferase (FLuc) and Renilla luciferase (RLuc) were measured using the DLR® Assay System (#E1960, Promega Corporation) according to the manufacturer's instructions on 10 μl of cell lysates using a Sirius Single Tube Luminometer (Berthold Detection Systems, GmbH, Pforzheim, Germany). Data are expressed as a percentage of the relative luciferase activity (RLA) or as relative translation activity (RTA). The RTA corresponds to the FLuc/RLuc ratio and is used as an index of the IRES activity (6,20,36).

#### RNA extraction and real-time RT-qPCR

HEK293T cells were seeded at 2.4 × 10^5^ cells per well in 12-well culture plates, and cytoplasmic RNA was extracted. Cells were washed twice with PBS 1× (#SH30256, Hyclone, GE Healthcare Life Sciences, Boston, MA, USA), and incubated 5 min on ice with RNLa buffer (10 mM Tris–HCl pH 8, 10 mM NaCl, 3 mM MgCl_2_, 0.5% NP-40, 1 mM DTT) containing 10 U of RNase inhibitor (#EO0381, Thermo-Fisher Scientific Inc.). After incubation, 500 μl of TRIzol reagent (#15596018, Life Technologies Corporation, Thermo Fisher Scientific Inc.) was added to the supernatant, and the RNA was recovered. Cytoplasmic RNA was resuspended in 25 μl of nuclease-free water, DNase-treated (#AM1907, Ambion, Thermo-Fisher Scientific Inc.), and recovered according to the manufacturer's instructions. The RNA concentration was quantified by nano-spectrophotometry (N60-Implen Nanophotometer, Westlake Village, CA, USA). The relative RNA quantification was carried out by real-time reverse transcription (RT)-quantitative polymerase chain reaction (qPCR) assay (QuantStudio 3 Real-Time PCR System, Thermo Fisher Scientific Inc.) using the Brilliant II SYBR Green RT-qPCR one Step Master Mix (#600835, Agilent Technologies, Santa Clara, CA, USA). Gag-RLuc RNA was amplified using primers Renilla sense (5′-AGGTGAAGTTCGTCGTCCAACATTATC-3′) and Renilla antisense (5′-GAAACTTCTTGGCACCTTCAACAATAGC-3′) as previously described (18). When required, to establish the amount of RLuc RNA, the Renilla sense and Renilla antisense primers were used. To determine the amount of FLuc RNA, the FLuc sense (5′-ACTTCGAAATGTCCGTTCGG-3′) and FLuc antisense (5′- GCAACTCCGATAAATAACGCG-3′) primers were used (38). No-RT-qPCR reactions were carried out to control for contaminant DNA Glyceraldehyde-3-phosphate dehydrogenase (GAPDH) mRNA was detected with the primers GAPDH sense (5′-TCCACCACCCTGTTGCTGTAG-3′) and GAPDH antisense (5′-ACCCACTCCTCCACCTTTGAC-3′) (45). Data analysis was performed by the ΔΔCt method as previously described in (46).

#### Cell viability

The cell viability assay was performed using the CellTiter 96® Aqueous One Solution Cell Proliferation Assay (MTS) (#G358A, Promega Corporation) according to the manufacturer's instructions. Briefly, HEK293T cells were seeded at 1.5 × 10^3^ cells per well in a 96-well plate and transfected with the indicated concentrations of hnRNPK (pCMV3-HA hnRNPK wt or mutants) expressing plasmids or the PRMT1 inhibitor TC-E 5003, the CellTiter 96® Aqueous One Solution Cell Proliferation Assay was added, incubated at 37°C for 4 h, and the absorbance was measured at 495 nm in a Biochrom EZ Read 400 microplate reader (Biochrom, Holliston, MA, USA).

#### Western blots

Cells were lysed using the Passive Lysis 5 × Buffer (#E1941, Promega Corporation). The concentration of total protein was determined by the Bradford assay using the Bio-Rad Protein Assay (#5000006, Bio-Rad Laboratories, Inc., Hercules, CA, USA). Equal amounts of protein (30 or 40 μg) were resolved by electrophoresis on a 10% or 12% glycine sodium dodecyl sulfate-polyacrylamide gel (SDS-PAGE) transferred onto a 0.45 μm nitrocellulose membrane (#10600002; GE Healthcare Bio-Sciences 100 ResultsWay, Marlborough, MA, USA). Membranes were blocked with Tris-buffered saline (pH 7.4) containing 5% skimmed milk and 0.1% Tween-20 (TBS-T) for 1 h at room temperature and incubated overnight at 4°C with the primary antibody. The membranes were washed three times with TBS-T and incubated with the corresponding horseradish peroxidase-conjugated secondary antibodies. The primary mouse anti-hnRNPK antibody (#sc-28380, Santa Cruz Biotechnology) was used at 1:500 dilution, a mouse anti-DDX3 (#ab50703, Abcam, Cambridge, UK), mouse anti-HuR (sc-5261, Santa Cruz Biotechnology), and a rabbit anti-p17 HIV-1 (#4811, NIH AIDS Reference and Reagents program) were used at 1:1000 dilution, a mouse anti-HA (#H9658, Sigma-Aldrich, 3050 Spruce Street, St. Louis, MO, USA), a mouse anti-GFP (#632381, Living Colors® A.v. Monoclonal Antibody JL-8, Clontech Laboratories, Inc., CA, USA), mouse anti-PARP-1 (sc-136208, Santa Cruz Biotechnology), mouse anti-hnRNPA1 (#sc-32301, Santa Cruz Biotechnology), and a mouse anti-GAPDH (#MA5-15738; Thermo Fisher Scientific Inc.) were used at a 1:5000 dilution. For stripping, membranes were incubated with stripping buffer (glycine 0.2 M, NaCl 0.5 M pH 2.8) for 15 min at room temperature, washed with TBS-T (Tris-buffered saline (pH7.4) 0.1% Tween-20) for 15 min at room temperature. The incubation and washing steps were repeated twice. Membranes were blocked with TBS-T containing 5% skimmed milk for 1 h at room temperature, washed three times with TBS-T, and incubated overnight at 4°C with the primary antibody. Either a Goat anti-mouse or Goat anti-rabbit IgG-horseradish peroxidase (HRP) conjugate (#AP308P, #AP132P; Merck, Darmstadt, Germany) secondary antibodies, both at 1:10 000 dilution, were used. The Asymmetric Di-Methyl Arginine (aDMA) Motif [adme-R] MultiMab™ Rabbit mAb mix (#13522, Cell Signaling Technology, Danvers, MA, USA) used at a 1:1000 dilution. Western blots were visualized by enhanced luminescence by a chemiluminescence reaction using 4-hydroxycinnamic acid (#800237, Merck) and luminol (#09253, Sigma-Aldrich), the SuperSignal™ West Femto Maximum Sensitivity Substrate (#34096, Thermo Fisher Scientific Inc.) or Western Lightning Plus-ECL (# NEL 121001, PerkinElmer Health Science Canada, Inc, Ontario, Canada). The western blot films (Fuji medical X-ray film Super HR-U 30) or Hyblot CL (# DV-3012, Denville Scientific Inc., NJ, USA)) were digitized using a CanonScan 9950F scanner or captured using an Alliance 2.7 imaging system (UVItec Cambridge, Topac Inc., 231 CJC Highway, Cohasset, MA, USA) (21,22).

### Co-immunoprecipitation (CoIP)

For the CoIP assays, 3 × 10^6^ HEK 293T cells were transfected with the dl HIV-1 IRES plasmid together with pCMV-HA-hnRNPK or pCMV-HA-hnRNPK-5RK plasmids. 48 hpt, cells were washed three times with PBS 1X and resuspended in lysis buffer (NaCl 100 mM, EDTA 2mM, Tris–HCl 50 mM pH 7.5, Naf 50 mM, sodium orthovanadate 1 mM, Triton X-100 1%, and protease inhibitors (#11836170001, Roche Diagnostics, Sigma-Aldric). The protein concentration in lysates was determined by Pierce BCA Protein assay (#23227, Thermo Fisher Scientific Inc). Protein A/G Plus-Agarose (#SC-2003, Santa Cruz Biotechnology) beads were incubated with the anti-HA mouse (#H9658, Sigma-Aldrich) or anti-IgG mouse antibody (#SC-2025, Santa Cruz Biotechnology) for 4 h. Beads were washed once with lysis buffer to discard unbound antibodies. Then 0.5 mg of total protein was incubated with the antibody-coated beads for 16 h at 4°C in rotation. The beads were washed with lysis buffer three times, glycine loading buffer (5×) was added, and the mix was heated (95°C for 5 min). The supernatant was recovered by centrifugation and subjected to western blot assay (as described above) using protein A/G conjugated with HRP (# 32490, Thermo Fisher Scientific Inc.) as the secondary antibody.

#### Immunofluorescence and microscopy

HeLa cells were seeded at 7 × 10^4^ cells per well on a 12-well plate previously prepared with sterilized cover glasses of 0.15 mm thickness (#16004-300, V.W.R. VistaVision). 24 h later, cells were transfected with the HIV-1-coding plasmid pNL4.3 or the empty vector pcDNA3.1, using JetPRIME® (#101000027, Polyplus, Illkirch, France) according to the manufacturer's recommendations. 24- or 48 hpt, coverslips were washed twice with 1X D-PBS and fixed in 4% PFA for 15 min, followed by 0.1M glycine for 10 min at room temperature. Cover glasses were blocked in a 1% BSA solution diluted in 1X D-PBS for 45 min at room temperature. For permeabilization, coverslips were incubated in a 0.2% Triton X-100 solution in 1× D-PBS for 5 min and washed before blocking. For mild permeabilization, 0.025% Saponin was added to the blocking buffer and kept during blocking and antibody incubation. After blocking, cells on cover glasses were incubated for 1 h at 37°C with a cocktail of primary antibodies: rabbit anti-p24 (#ARP-4250, NIH HIV reagent program) and mouse anti-hnRNPK (#Ab39975, Abcam) or mouse anti-hnRNPA2 (hybridoma EF67 (47), generously provided by Dr. William Rigby, Dartmouth Medical School, NH USA), all used at 1:100 dilution. Cover glasses were washed four times in 1× D-PBS for 5 min and incubated as before with a cocktail of secondary antibodies (Invitrogen donkey anti-rabbit-AlexaFluor 488 [A21206], and donkey anti-mouse-AlexaFluor 594 [A21203], each used at 1:300 dilution). The washing protocol was repeated, and cover glasses were incubated with DAPI (4',6-Diamidino-2-Phenylindole, Dihydrochloride) (#D1306, Invitrogen), for 10 min. Cover glasses were dried at room temperature and then mounted using a drop of Immu-Mount (#9990402, Thermo Fisher Scientific Inc). Images were acquired using the Zeiss LSM800 confocal microscope using immersion oil in a 40×/1.4 numerical aperture objective). Images were processed and analyzed in Imaris v10.0.0 (Bitplane, Andor Inc., Oxford Instruments, UK).

HEK293T cells were seeded at 2.5 × 10^4^ cells per well in a 24-well culture plate at 60% confluence, previously prepared with sterilized cover glasses of 0.12 mm thickness, then transfected with 200 ng of the pSP64 poly(A), pCMV-HA-hnRNPK or pNL4.3-Rluc plasmids. 48- or 72-hpt, depending on the treatment, the cells were washed with PBS 1× and fixed with 4% paraformaldehyde (PFA) for 10 min. Then, the cells were permeabilized with PBS-Tr (PBS 1X, Triton X-100 0.03%) and blocked with 10% BSA in PBS-Tr for 1 h. Cover glasses was incubated with primary antibodies in 5% BSA in PBS-Tr overnight at 4°C 16 h, mouse anti-hnRNP K antibody (sc-28380) was used at dilution 1:100, rabbit anti-HA (#H6908; Sigma-Aldrich), goat anti-eIF3 (sc-16377), and rabbit anti-p24 were used at dilution 1:300. 24 h later the cover glasses were washed 5 times with PBS-Tr and incubated at room temperature for 2 h with a cocktail of secondary antibodies (Invitrogen; donkey anti-rabbit-AlexaFluor 647 [A31573] and donkey anti-mouse-AlexaFluor 488 [A21202], each diluted 1:300). The cover glasses were washed 3 times with PBS-Tr, once with PBS, and once with ultrapure water and incubated with Vectashield H1200 (Vector Laboratories, Inc, Burlingame, CA 94010, USA) with 4,6-diamidino-2-phenylindole (DAPI) as a mounting medium, sealed with clear nail polish and stored at 4°C. The images were captured with 40× or 63× magnification using a ZEISS microscope, axio observer D.1 model, and processed with the ImageJ program.

#### UV-CLIP

HEK 293T cells (9.0 × 10^6^) were transfected with the dl HTLV-1 IRES, dl sHBZ IRES or dl ΔEMCV IRES, and the HA-hnRNP K encoding vector. 24 hpt cells were washed, covered with cold PBS 1X (7mL/10 cm plate) (#SH30246.01, HyClone), and UV-irradiated (UV-254 nm, 400 mJ/cm^2^) on ice in a UV chamber (UVP, UV Crosslinker CL-1000, Upland, CA, USA). Cells were scraped, collected by centrifugation at 4ºC, and lysed using RIPA buffer (Tris-HCl pH 7.5 10mM, EDTA 1mM, NaCl 150mM, NP-40 0.5%, Sodium deoxycholate 0.5%, SDS 0.1%, and protease inhibitors (Roche Diagnostics), 3 μL Riboblock (#EO0381, Thermo Fisher Scientific Inc) or SUPERase•In™ (#AM2694, Ambion, Life Technologies) and 6 μl DNAseRQ1 (#M6101, Promega Corporation), and sonicated (Sonic Ruptor 250, Omini International The homogenizer Company, Kennesaw, GA, USA) on ice. The total lysate was precleared with protein A/G coated beads (#SC-2003, Santa Cruz Biotechnology) for 1 h in rotation at 4°C and centrifugated. The protein concentration of the supernatant was determined using the Pierce™ B.S.A. assay (#23227,Thermo Fisher Scientific Inc). 300 μl of cell lysate was saved as input for RNA and protein extraction, 800 μl (1.5 mg of total proteins) was allocated for RNA extraction, and 500 μl (1 mg total protein) of the lysate was used for western blots. For immunoprecipitation assay (IP), protein extracts were mixed with protein A/G beads loaded with an anti-HA monoclonal antibody. After 16 h at 4ºC, with rotation. For samples designated to western blots, the beads were mixed with tricine loading buffer (2X) and heated (90ºC for 10 min), loaded and resolved in a tris-tricine gel, and transferred to a 0.45 μm PVDF membrane (#88518, Thermo Scientific Inc), the anti-HA primary antibody was used. The recombinant protein A/G conjugated with HRP was used as the secondary antibody (#32490, Thermo Fisher Scientific Inc). For RNA extraction, the beads were washed with Buffer PK (0.05M Tris-HCl pH 8, 0.5M LiCl, 0.03M EDTA, 0.5% SDS, 0.1% ß-mercaptoethanol and Proteinase K (Invitrogen)) and with Buffer PK + urea 7 M. Then 500 μl of TRIzol (#15596018, Life Technologies Corporation) was added, and RNA extraction proceeded as suggested by the manufacturer. The total RNA concentration was quantified using a nano-spectrophotometer (N60-Implen, Nanophotometer, CA, USA). RT-qPCR assays to detect FLuc and GAPDH mRNA were performed as described above. Fold Enrichment (FE) of FLuc encoding RNA was calculated as 2^(−ΔΔCt [HA/IgG])^ as detailed in (46).

#### Subcellular fractionation

HEK293T cells (2.5 × 10^6^) were seeded in 90 mm culture plate, and cells at 70–80% confluence were transfected with 4.2 μg of plasmids pCMV3-HA-HNRNPK or pSP64 Poly(A) according to the assay. 24 hpt, the culture medium was removed, and the cells were resuspended in 1 ml of PBS 1×, then centrifuged at 1000 × g for 3 min at 4ºC. The pellet was resuspended in 1 ml of PBS 1×; 0.3 ml was used for the preparation of the complete cell extract, and 0.7 ml was used to obtain the nuclear and cytoplasmic fractions. For the preparation of the complete cell extract, the cells were centrifuged at 1000 × g for 5 min at 4°C. The pellet was resuspended in 300 μl of RIPA buffer (Tris–HCl pH 7.5 10 mM, EDTA 1mM, NaCl 150 mM, NP-40 0.5%, sodium deoxycholate 0.5%, SDS 0.1% and protease inhibitor (Roche Diagnostics)) and then sonicated on ice at 40% amplitude for 20 s. The total cell extracts were stored at –20ºC. Cells were centrifuged at 1000 × g for 5 min at 4°C to obtain the nuclear and cytoplasmic fractions. Then, the pellet was resuspended in 300 μl of buffer RLNa (10 mM of Tris–HCl (pH 8), 10 mM NaCl, 3 mM MgCl2, 1 mM DTT, 0.5% NP40, 10 U/μl of RNAse inhibitor (Thermo-Fisher Scientific Inc.)) and incubated on ice for 5 min. The lysate was centrifuged at 16 000 × g for 3 min. The supernatant obtained was recovered, centrifuged under the abovementioned conditions, and preserved as the cytoplasmic fraction. The pellet obtained was resuspended in 300 μl buffer RLNa and centrifuged at 16 000 × g for 3 min. The pellet was resuspended in 300 μl of RIPA buffer and sonicated on ice, and the lysate obtained was preserved as the nuclear fraction. The specificity of the preparation of the previously described extracts was evaluated by western blot assays, using anti-poly (ADP-ribose) polymerase (PARP) (nuclear) or anti-GAPDH (cytoplasmic) antibodies.

### Statistical analysis

Graphics and statistical analysis were performed using the Prism v9.5.1 software (GraphPad Software LLC, San Diego, CA, USA), completing the statistical test indicated in the text and figure legends.

## Results

### HnRNPK promotes HIV-1 Gag protein synthesis

**Figure 1.**
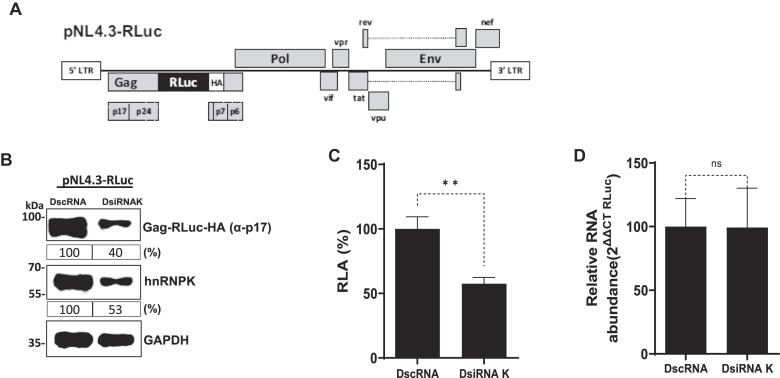
HnRNPK regulates HIV-1 Gag expression. (**A**) Schematic representation of the complete HIV-1 molecular clone pNL-4.3-RLuc. (**B**) HEK 293T cells were cotransfected with the pNL-4.3-RLuc (200 ng) plasmid and 10 nM of a DsiRNA targeting the hnRNPK mRNA (DsiRNAK) or with a scrambled RNA ((DscRNA); 10 nM) as a control. The expression of the HIV-1 Gag-RLuc-HA fusion protein and endogenous hnRNPK was determined 24 hpt by western blot using the GAPDH protein as a loading control. For the semi-quantitative comparative analysis, the captured images were quantified using the ImageJ 1.53 software (Windows version of NIH ImageJ, http://imagej.nih.gov/ij). Values expressed in percentages (%) correspond to the ratio (OD value (p17 or hnRNPK)*/*OD value GAPDH) relative to the control (DscRNA) set to 100%. (**C**) *Renilla* luciferase activity was measured 24 hpt and is expressed as relative luciferase activity (RLA) relative to the activity obtained when the pNL-4.3-RLuc plasmid was cotransfected with the DscRNA(−) set to 100%. (**D**) Cytoplasmic RNA was extracted from cells expressing pNL-4.3-RLuc HIV-1 and treated with the DscRNA or the DsiRNAK (10 nM), and relative RNA levels were determined by real-time RT-qPCR. The RNA abundance was expressed relative to the value obtained for the cells treated with the DscRNA set to 100%. In (**C**) and (**D**), values represent the mean (±SEM) from six independent experiments, with each conducted in duplicate. Statistical analyses were performed by an unpaired two-tailed *t*-test (ns = nonsignificant; ** *P* ≤ 0.01).

A proteomic screening assay using HeLa extracts as the source of proteins identified hnRNPK as a protein that interacts with the 5′UTR of the HIV-1 vRNA (clone NL4.3) (24), the region that harbors the HIV-1 IRES (4). We wondered whether hnRNPK was a regulator of HIV-1 protein synthesis. To address this question, we decided to use HEK293T cells, which, together with HeLa cells, are common tools used by us and others to study the molecular biology of HIV-1 (4,9,11,14,21,22,24). HEK293T cells were cotransfected with the HIV-1 clone NL-4.3-RLuc DNA together with a commercial Dicer-Substrate siRNA (DsiRNA), targeting the 3′UTR of the endogenous hnRNPK mRNA (DsiRNAK, 10nM), or with a non-related scrambled control (DscRNA, 10nM). The pNL-4.3-RLuc plasmid (Figure 1A) has a hemagglutinin (HA)-ta*gged Renilla* luciferase (RLuc-HA) reporter gene inserted in frame with the group-specific antigen (Gag)-protein start codon, generating a Gag-RLuc-HA fusion protein (35). Transfecting the HIV-1 DNA enables us to skip the highly regulated initiation steps in virus replication, including receptor recognition, entry, reverse transcription, and integration. Thus, we only focus on replication steps starting with viral RNA transcription. As expected, treating cells with DsiRNAK decreased the detectable levels of endogenous hnRNPK protein (Figure 1B). In agreement with a previous report (28), the decrease of endogenous hnRNPK levels reduced the amount of HIV-1 Gag-RLuc-HA protein, detected by using an antibody against Gag (anti-p17) (Figure 1B). Consistent with lower viral protein levels (Figure 1B), a significant (*P*< 0.05) decrease in RLuc activity ($\sim$43% reduction) in cells treated with the DsiRNAK was also evidenced (Figure 1C). Cytoplasmic RNA was extracted from pNL-4.3-RLuc (DNA), DscRNA, or DsiRNAK cotransfected cells and used as a template for quantitative analysis of the NL-4.3RLuc RNA by RT-qPCR. The relative levels of NL-4.3-RLuc vRNA were equivalent in the DscRNA- and DsiRNAK-treated cells (Figure 1D). Thus, the decrease in Gag-RLuc-HA levels in HEK293T cells treated with the DsiRNAK RNA (Figure 1B and C) was not associated with a reduction in the abundance of HIV-1 vRNA (Figure 1D). These results indicate that a decline in endogenous hnRNPK reduces HIV-1 vRNA translation, suggesting that hnRNPK plays a role in HIV-1 protein synthesis.

### HIV-1 gene expression does not induce a shift of hnRNPK subcellular distribution

**Figure 2.**
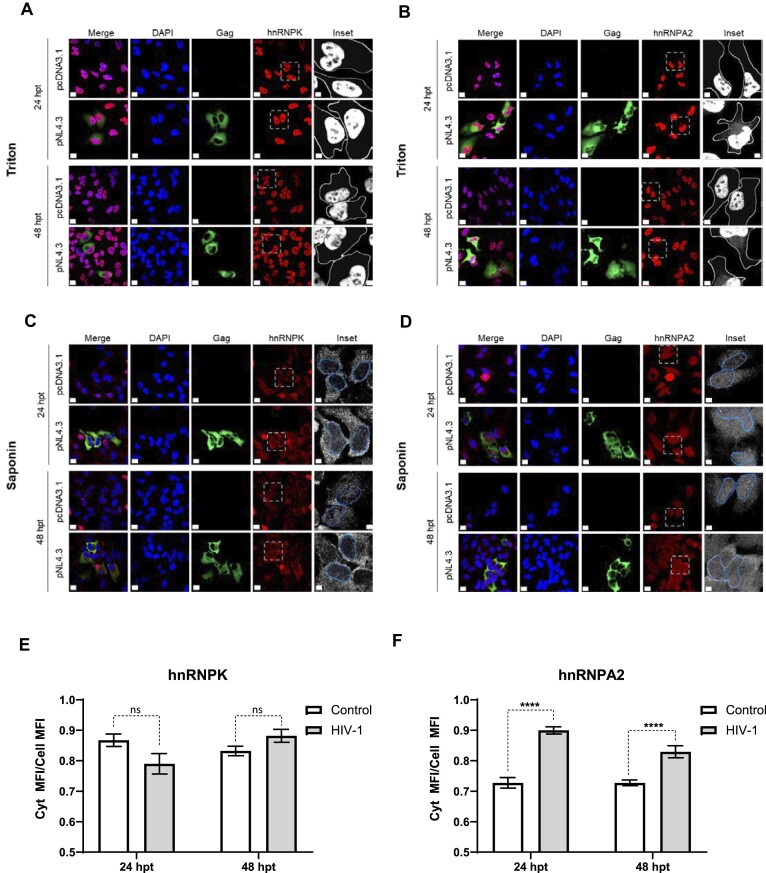
HIV-1 gene expression does not induce hnRNPK localization from the nucleus to the cytoplasm. HeLa cells were transfected with pNL4.3 or the control vector pcDNA3.1. 24- and 48-hpt, cells were fixed and permeabilized using Triton X-100 (strong detergent) (**A, B**) or saponin (mild detergent) (**C, D**). HeLa cells were stained against HIV-1 Gag (green) and hnRNPK (red) (**A, C**) or hnRNPA2 (red) (**B, D**). An overexposed closer magnification on the red channel, hnRNPK (**A, C**) or hnRNPA2 (**B, D**), is shown in the right column (Inset), where gray outlines the plasma membrane and blue the nucleus. Scale bar = 15 μm (Inset = 5 μm). (**E, F**) hnRNPK and hnRNPA2 were quantified in saponin-permeabilized cells by determining the mean fluorescence intensity (MFI) ratio between the cytoplasmic MFI and the overall cell MFI in the hnRNPK or hnRNPA2 channel. Individual cell values were obtained by using the cell detection function of the Imaris v10.0 software. Each bar represents the mean value (±SEM) from 20 to 30 cells per condition. Statistical analyses were performed by multiple *t*-tests between the control and the HIV-1 condition per group. Statistical significance was determined using the Holm–Sidak method (**** *P* ≤ 0.0001).

HnRNPK is a nucleo-cytoplasmic shuttling protein mainly concentrated in the nucleus (48). HIV-1 replication does not upregulate hnRNPK expression levels in infected cells (49). However, HIV-1 replication may alter the protein's subcellular distribution, as evidenced for hnRNPA2 (50). Thus, we wondered whether HIV-1 gene expression induces a shift in endogenous hnRNPK cellular compartmentalization. Therefore, HeLa cells were transfected with pNL-4.3 DNA or control vector DNA, pcDNA3.1, and hnRNPK localization was analyzed by indirect immunofluorescence (IF). HIV-1 expression was monitored by detecting Gag protein in cells at 24 and 48 hpt along with endogenous hnRNPK (Figure 2A and C) or hnRNPA2 as a positive control (Figure 2B and D). Because of the nuclear abundance of hnRNPK, cells were permeabilized using a strong (Triton X-100) or a mild (saponin) agent, as Triton X-100 enhances signals from nuclear staining, while saponin enhances the cytoplasmic fluorescence signal (51,52). As expected for a nucleo-cytoplasmic shuttling protein, in HeLa cells, hnRNPK was detected in both the cell cytoplasm (saponin) and the nuclear (Triton X-100) compartments (Figure 2A and C). In agreement with an earlier report (48), hnRNPK localization was mainly nuclear. At 24 or 48 hpt of the pNL4.3 DNA, hnRNPK, and non-significant increasing trend of cytoplasmic hnRNPK was observed, but most remained nuclear while HIV-1 Gag protein was cytoplasmic (Figure 2A, C, and E). Under similar experimental conditions, a fraction of hnRNPA2 was repositioned to the cytoplasm (Figure 2B, D and F) (50). Similarly, in HIV-1 expressing HEK293T cells, endogenous hnRNPK remained mainly nuclear (Supplemental Figure S1). Thus, we conclude that in HeLa and HEK293T cells, HIV-1 gene expression does not induce a detectable redistribution of endogenous hnRNPK to the cell cytoplasm.

### HnRNPK participates in HIV-1 IRES-mediated translation in cells

**Figure 3.**
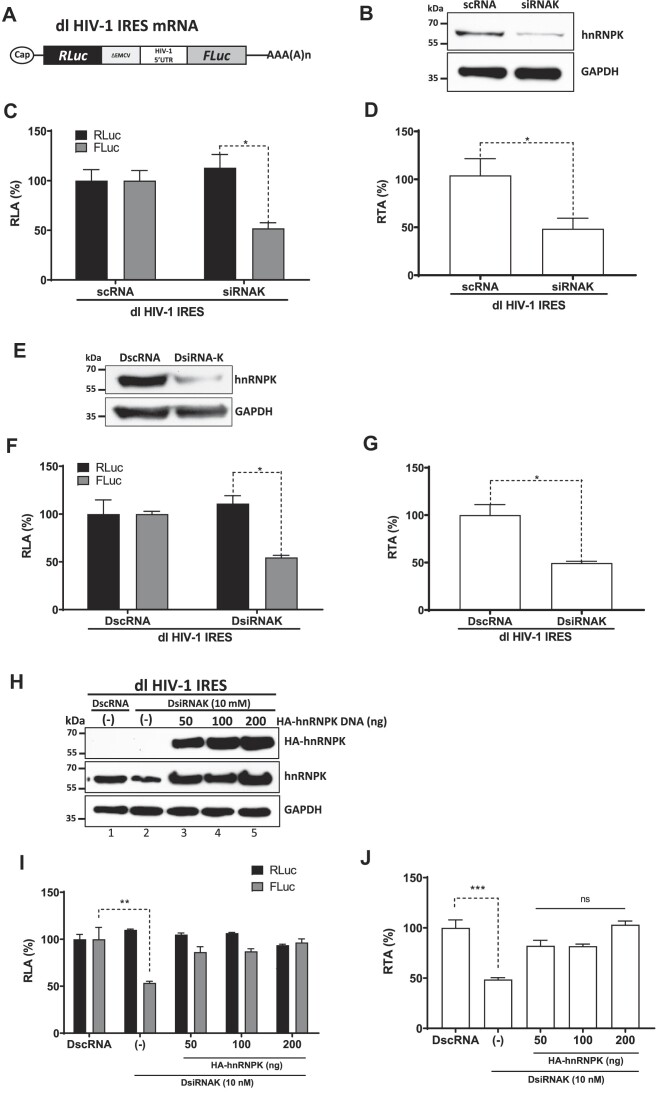
Reduction of endogenous hnRNPK levels in cells negatively impacts HIV-1 IRES activity. (**A**) Schematic representation of dl HIV-1 IRES mRNA. The capped and polyadenylated dual-luciferase (dl) bicistronic mRNA presents an upstream *Renilla* luciferase (RLuc) ORF and a downstream firefly luciferase (FLuc) ORF. A deleted 5′UTR of the encephalomyocarditis virus (ΔEMCV) followed by the 5′UTR (nucleotides 1-336) of the HIV-1 vRNA, NL-4.3 clone, are placed between both cistrons. (**B–G**) HEK293T cells were cotransfected with the dl HIV-1 IRES (200 ng) mRNA encoding plasmid, a scRNA (200 nM), or a siRNAK (**B-D**) targeting hnRNPK or with a DscRNA (10 nM) control or DsiRNAK (E–G). Reduction of endogenous hnRNPK was monitored by western blot using GAPDH as a loading control (**B** and **E**). RLuc and FLuc activities were measured at 24 hpt, and data are presented as RLA (**C** and **F**) or as RTA (**D** and **G**). RTA corresponds to the FLuc/RLuc ratio that is used as an index of IRES activity. The RLA and RTA values obtained in the absence of HA-hnRNPK plasmid were set to 100%. (**H–J**) HEK293T cells were transfected with the dl HIV-1IRES plasmid with an irrelevant control DNA (200 ng) and DsiRNAK (10 mM) alone or with increasing concentrations (50-200 ng) of a plasmid encoding for a HA-hnRNPK. The levels of endogenous and overexpressed hnRNPKs were monitored by western blot (**H**). RLuc and FLuc activities were measured at 24 hpt, and data are presented as RLA (**I**) or as RTA (**J**). The RLA and RTA values obtained in cells transfected with the dl HIV-1 IRES plasmid and the DscRNAK RNA were set to 100%. Values represent the mean (±SEM) from three independent experiments, with each conducted in duplicate. Statistical analysis was performed by performed for (**C, D**) by Student's *t*-test (* *P*< 0.05) and (**F, G**) and ordinary one-way ANOVA test (** *P* < 0.005, **** *P* < 0.0001; ns, not significant)

Next, we assessed whether hnRNPK acted as an ITAF for the HIV-1 IRES. Based on our earlier studies (4,18,20,21,36), we used the well-characterized dual-luciferase (dl) reporter plasmid dl HIV-1 IRES that encodes for a bicistronic mRNA with an upstream *Renilla* luciferase (RLuc) ORF and a downstream firefly luciferase (FLuc) ORF (Figure 3A) (4). Placed between both cistrons, the dl HIV-1 IRES RNA has a deleted 5′UTR of the *encephalomyocarditis virus* (ΔEMCV) RNA, a highly structured element deficient in IRES activity that impedes ribosome reinitiation and read-through, followed by the 5′UTR (nucleotides 1-336) of the HIV-1 vRNA (NL-4.3 clone) (4). First, we sought to determine if endogenous hnRNPK modulates the activity of the HIV-1 IRES. For this, HEK293T cells were transfected with the dl HIV-1 IRES plasmid, a scrambled (sc) RNA (200 nM) or DscRNA (10 nM) control, or a siRNAK (Figure 3B–D) or DsiRNAK (Figure 3E–G). Western blot analysis confirmed that treating cells with either the siRNAK or DsiRNAK reduced hnRNPK (Figure 3B and E). As an aggressive knockdown of hnRNPK can induce cell death (53), we decided to verify cell viability. The results showed that the level of reduction in hnRNPK induced by either the siRNAK or DsiRNAK did not affect cell viability (Supplemental Figure S2A). Luciferase activities were then measured and expressed as relative luciferase activity (RLA), with the RLuc and FLuc levels from cells transfected with the negative controls, scRNA or DscRNA, set to 100% (Figure 3C and F). A significant decrease ($\sim$48%) of FLuc with a slight increase of RLuc ($\sim$13%) was observed in cells transfected with the dl HIV-1 IRES plasmid and treated with the siRNAK (Figure 3C). Similar observations were made when the DsiRNA was used (Figure 3E), with a slight increase in RLuc ($\sim$11%) activity and a significant decrease in FLuc activity ($\sim44$%). As RLuc activity did not decrease, these results suggest that the reduction of FLuc activity when hnRNPK is targeted by either the siRNAK or the DsiRNAK cannot be attributed to the reduced stability of the dl HIV-1 IRES mRNA (Figure 3C and F). Analysis of the FLuc/RLuc ratio (relative translational activity, RTA), confirmed the significant reduction in HIV-1 IRES activity in cells treated with either siRNAK ($\sim$52%) or DsiRNAK ($\sim$51%) (Figure 3D and G).

To further validate the association between HIV-1 IRES activity and hnRNPK levels, HEK293T cells were transfected with the dl HIV-1IRES plasmid with the DsiRNAK (10 mM) RNA and an irrelevant control DNA (200 ng) or with increasing concentrations (50–200 ng) of a plasmid encoding a hemagglutinin (HA_3_)-tagged hnRNPK, HA-hnRNPK. The HA-hnRNPK mRNA lacks the hnRNPK mRNA 3′UTR and, therefore, is not susceptible to DsiRNAK RNA. As expected, treatment of cells with the DsiRNAK RNA reduced endogenous hnRNPK levels (Figure 3H, lane 2), also RLuc activity slightly increased ($\sim$10%), while FLuc activity decreased significantly ($\sim$46%) (Figure 3I). In cells expressing the DsiRNAK RNA and HA-hnRNPK (Figure 3H, lanes 3–5), FLuc activity was restored (Figure 3I). When data are presented as RTA, DsiRNAK treatment led to reduced HIV-1 IRES activity ($\sim$51%) in cells, but HIV-1 IRES activity was restored when the hnRNPK expression was rescued (Figure 3J). Thus, we conclude that HA-hnRNPK promotes HIV-1 IRES activity in HEK293T cells.

### HnRNPK overexpression stimulates HIV-1 IRES activity

**Figure 4.**
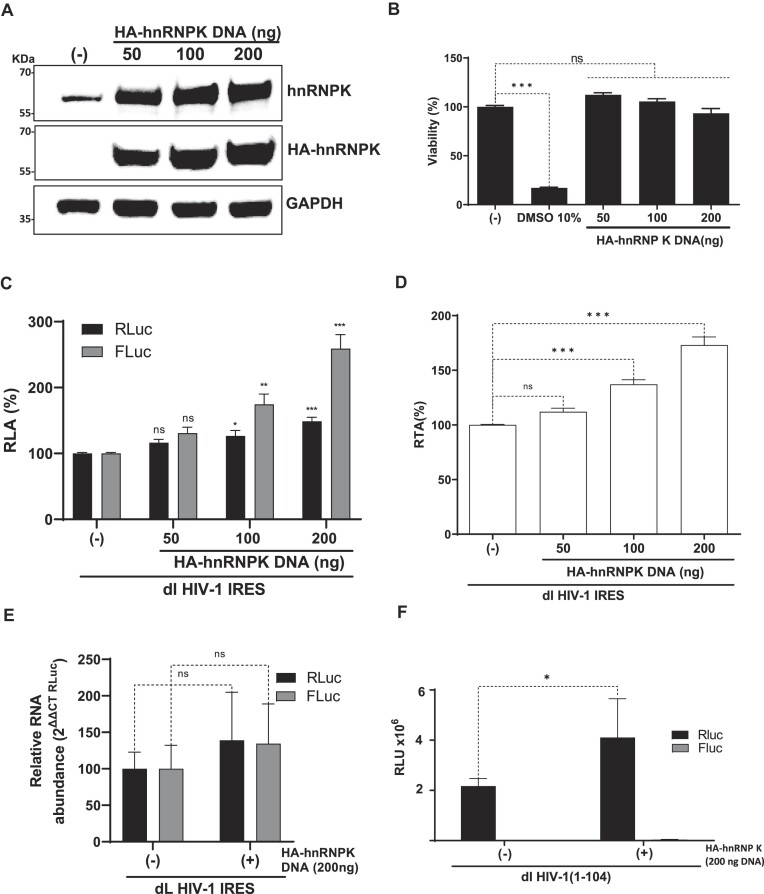
Overexpression of hnRNPK promotes HIV-1 IRES activity. (A-D) HEK293T cells were cotransfected with the dl HIV-1 IRES (200 ng) and different quantities (50-200 ng) of a plasmid encoding for an HA-hnRNPK protein. (**A**) The presence of the endogenous hnRNPK and overexpressed HA-hnRNPK proteins was confirmed by western blot using GAPDH as a loading control. (**B**) Cell viability was determined by measuring the cellular metabolic activity using the MTS assay using dimethylsulfoxide (DMSO, 10%) as a control for cell death. Data are expressed relative to the viability of the cells transfected with control DNA (−) set to 100%. Values shown are the mean (±SEM) from three independent experiments, with each performed in duplicate. Statistical analysis was performed by an ordinary one-way ANOVA test (*** *P*< 0.001). (**C, D**). RLuc and FLuc activities were measured at 24 hpt, and data are presented as RLA (**C**) or as RTA (**D**). The RLA and RTA values obtained in the absence of HA-hnRNPK plasmid were set to 100%. Values shown are the mean (±SEM) from four independent experiments, with each performed in duplicate. Statistical analysis was performed using ANOVA, followed by Dunnet's test (*P≤ 0.05; **P≤ 0.01; ***P≤ 0.001; ns = nonsignificant). (**E**) Cytoplasmic RNA was extracted from cells transfected with dlHIV-1 IRES and pCMV3-HA-hnRNPK or with the control plasmid, pSP64-polyA. The relative amount of RNA was determined by RT-qPCR assay. The relative RNA abundance was obtained by setting the value obtained from the cells transfected only with pSP64-polyA as 100% for both RLuc and FLuc. Values represent the mean (±SEM) from four independent experiments, with each performed in duplicate. Statistical analyses were performed by an unpaired two-tailed *t*-test (ns = nonsignificant). (**F**) HEK293T cells were cotransfected with the dl HIV-1 (1–104) DNA and a plasmid (200 ng) encoding for a HA-hnRNPK protein. RLuc and FLuc activities were measured 24 hpt, and data are presented as relative light units (RLU). Values shown are the mean (±SEM) from three independent experiments, with each condition performed in duplicate. Statistical analysis was performed using ANOVA, followed by Dunnet's test (*P≤ 0.05).

Next, HEK293T cells were transfected with the dlHIV-1 IRES plasmid, an irrelevant DNA (negative control), or different concentrations (50, 100 or 200 ng) of the HA-hnRNPK plasmid. The overexpression of HA-hnRNPK was monitored by western blot using an anti-hnRNPK or an anti-HA antibody, using GAPDH as a loading control (Figure 4A). The overexpression of HA-hnRNPK did not appreciably impair cell viability (Figure 4B). Luciferase activities were measured, and data were expressed as RLA, with the values of the luciferase activities obtained from cells transfected with the control DNA (–) set to 100% (Figure 4C). A dose-dependent increase in FLuc activity with increasing HA-hnRNPK (50–200 ng of DNA) was observed (Figure 4C). While there was little impact on RLuc activity at 50 ng HA-hnRNPK DNA, with a significant increase of RLuc with 100 ng HA-hnRNPK DNA, the increase in RLuc activity was highest when 200 ng HA-hnRNPK was transfected in cells (Figure 4C). Nonetheless, the increase in RLuc activity was lower than that of FLuc, as evidenced when the FLuc/RLuc ratio (RTA) is analyzed (Figure 4D).

The increase in RLuc and Fluc could be due to the stabilization of the HIV-1 IRES RNA. To evaluate this possibility, total RNA was extracted, and the relative amount of RLuc and FLuc encoding RNA in the presence or the absence of HA-hnRNPK, was independently determined. Results from these analyses show that the presence of HA-hnRNPK did not induce a significant change in RNA either determined by RLuc (39%) of FLuc (34%) RNA quantification (Figure 4E). Thus, changes to the stability of the dl HIV-1 IRES RNA cannot explain the increase of FLuc over RLuc protein expression. These results confirm that the overexpression of hnRNPK stimulates HIV-1 IRES activity from the dl HIV IRES RNA.

As an additional control, we assessed the impact of HA-hnRNPK on RLuc and FLuc expression from vector dl HIV-1 (1–104), which harbors the upstream sequence of the HIV-1 vRNA 5′UTR (nts 1–104) that is devoid of IRES activity (4). Plasmid dl HIV-1 IRES and dl HIV-1 (1–104) only vary in the segment of 5′UTR of the HIV-1 within the intercistronic space (4). The overexpression of hnRNPK significantly increased the expression of RLuc ($\sim$89%) but did not correlate with an increase in FLuc activity (Figure 4F). These results confirm that the expression of RLuc and FLuc are independent and that upstream events leading to an increased RLuc expression do not contribute to FLuc expression. Furthermore, these results confirm that an active IRES is required to generate FLuc from the dl HIV-1 IRES mRNA.

### The overexpression of HnRNPK does not enhance cryptic promoter activity from the dl HIV-1 IRES DNA nor induces alternative splicing from the dl HIV-1 IRES RNA in cells

**Figure 5.**
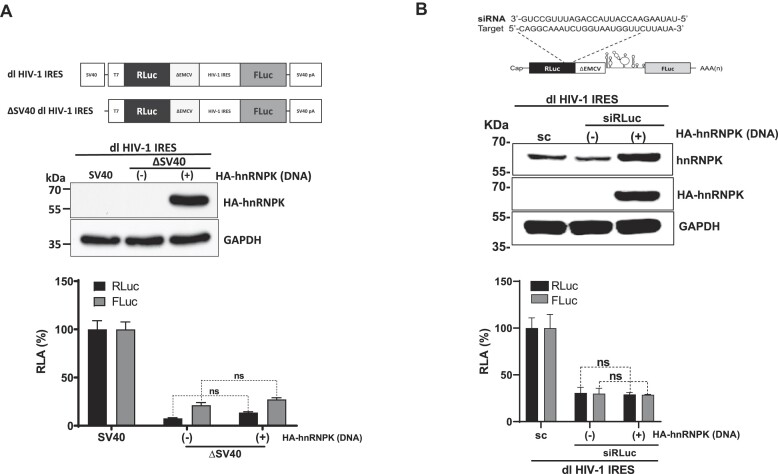
Overexpression of hnRNPK does not enhance cryptic promoter activity of the dl HIV-1 DNA or induce alternative splicing of the dl HIV-1 IRES RNA. (**A**) HEK 293T cells were transfected with either the dl HIV-1 IRES (150 ng) or a promoterless ΔSV40-dl HIV-1 IRES (150 ng) vector in the presence or the absence (−) of the HA-hnRNPK (100 ng) plasmid. 24 hpt total protein extracts were prepared. A schematic representation of the dl HIV-1 IRES and ΔSV40-dl HIV-1 IRES plasmids is shown (upper panel). The expression of the HA-hnRNPK recombinant protein was determined by western blot, using the GAPDH protein as a loading control (middle panel). RLuc and FLuc activities were measured, and results are expressed as RLA relative to the activities obtained from the dl HIV-1 IRES vector when in the absence of the HA-hnRNPK, set to 100% (lower panel). Values shown are the mean (±SEM) from three independent experiments, with each performed in duplicate. Statistical analysis was performed by an ordinary two-way ANOVA test (****P< 0.0001; ns, nonsignificant). (**B**) The dl HIV-1 IRES (200 ng) was cotransfected with a control scRNA (50 nM) or with siRLuc (50 nM), in the presence or the absence (−), of the HA-hnRNPK (200 ng) plasmid. A schematic representation of the dl reporter targeted by the siRNA RLuc (siRLuc) targeting the *Renilla* luciferase ORF is shown (upper panel). Total protein extracts were prepared 48 hpt. The expression of the HA-hnRNPK was determined by western blot, using the GAPDH protein as a loading control (middle panel). RLuc and FLuc activities were measured and expressed relative to the values obtained with scRNA, set to 100% (RLA) (lower panel). Values shown are the mean (±SEM) from three independent experiments, with each performed in duplicate. Statistical analysis was performed by an ordinary one-way ANOVA test (*P< 0.05; ns, nonsignificant).

The dl HIV-1 IRES reporter plasmid displays cryptic promoter activity in HEK293T cells (21,22). Therefore, we sought to determine if the overexpression of hnRNPK increased cryptic promoter activity within the dl HIV-1 IRES plasmid in HEK293T cells. For this, cells were transfected with the dl HIV-1 IRES or the ΔSV40 dl HIV-1 IRES plasmid in the presence or absence of HA-hnRNPK. The expression of HA-hnRNPK was monitored by western blotting of total cell lysates using antibodies directed against the HA-tag and GAPDH as a loading control (Figure 5A). In the absence of the SV40 promoter (ΔSV40; (Figure 5A) and (21,22)), RLuc and FLuc activities from the reporters were significantly (*P*< 0.05) diminished in cells (Figure 5A). In agreement with earlier reports (21,22), RLuc activity was detected in HEK293T, confirming both the leakiness of the experimental system and the previously reported weak cryptic promoter activity of the dl-plasmid in HEK293T cells (21,22). The presence of HA-hnRNPK did not further modulate RLuc or FLuc expression from the ΔSV40 dl HIV-1 IRES plasmid, indicating that the overexpression of HA-hnRNPK did not enhance cryptic promoter activity from the dl HIV-1 IRES plasmid in HEK293T cells (Figure 5A).

HnRNPK is involved in HIV-1 pre-mRNA splicing (27). To evaluate if the overexpression of HA-hnRNPK induced an alternative splicing event leading to the synthesis of a FLuc encoding monocistronic mRNA, the *Renilla* ORF of the dl HIV-1 IRES mRNA was targeted with a short interfering RNA, siRLuc (Figure 5B, upper panel) (21,22). Alternatively, HEK293T cells were cotransfected with the dl HIV-1 IRES and a control scRNA (Figure 5B). The expression of HA-hnRNPK was confirmed by western blot analyses using an anti-HA or anti-hnRNPK antibody and GAPDH as a loading control (Figure 5B, middle panel). In the presence of the siRLuc RNA, both RLuc and FLuc activities were significantly reduced, whether HA-hnRNPK was overexpressed or not (Figure 5B, lower panel). When directly compared, the reduction of RLuc and FLuc activities induced by the siRLuc RNA in the presence or absence of overexpressed HA-hnRNPK protein was not statistically different (Figure 5B, lower panel). This observation confirms that the overexpression of HA-hnRNPK did not favor the generation of a FLuc-expressing monocistronic transcript from the dl HIV-1 IRES RNA.

### ITAF activity of hnRNPK is modulated by post-translational modification

**Figure 6.**
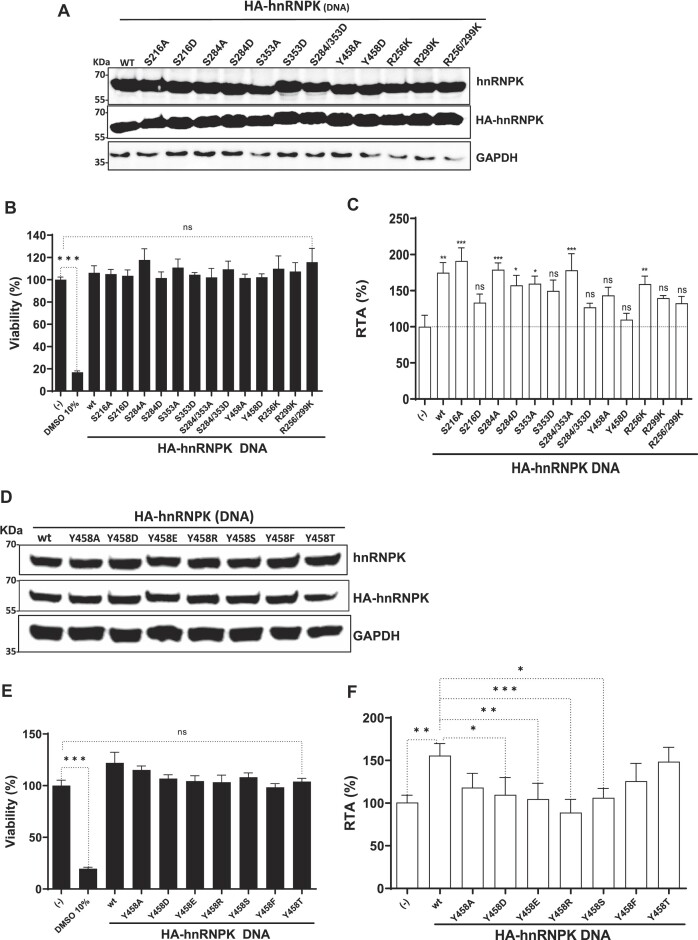
hnRNPK post-translational modifications impact on HIV-1 IRES activity. HA-hnRNPK mutants (mut) for the phosphorylation or methylation sites were generated by site-directed mutagenesis of the HA-hnRNPK template plasmid (wt). HEK293T cells were cotransfected with the dl HIV-1 IRES (200 ng) together with each HA-hnRNPK (200 ng) plasmid (indicated in the X-axis). Total protein extracts were prepared 24 hpt. (**A**) Western blots were performed to detect the expression level of total hnRNPK and HA-hnRNPK proteins using GAPDH as a loading control. (**B**) Cell viability was determined by measuring the cellular metabolic activity using the MTS assay using dimethylsulfoxide (DMSO, 10%) as a control for cell death. Data are expressed relative to the viability of the cells transfected with control DNA (−) set to 100%. Values shown are the mean (±SEM) from three independent experiments, with each performed in duplicate. Statistical analysis was performed using ANOVA, followed by Dunnet's test (***P≤ 0.001; ns = nonsignificant). (**C**) RLuc and FLuc activities were measured, and results are presented as RTA, relative to the activities in the presence of the HA-hnRNPK (wt), set to 100%. Values shown are the mean (± SEM) from five independent experiments, with each performed in duplicate. Statistical analysis was performed using ANOVA, followed by Dunnet's test (*P≤ 0.05; **P≤ 0.01; ***P≤ 0.001; ns, nonsignificant). (D–F) HEK293T cells were cotransfected with dl HIV-1 -IRES plasmid (200 ng) and a plasmid expressing HA-hnRNPK(wt) or the Y458 mutants (200 ng). (**D**) Proteins were monitored by western blot analysis using GAPDH as a loading control. (**E**) Cell viability was determined as in (B); values are the mean (±SEM) from three independent experiments, with each performed in duplicate. Statistical analysis was performed using ANOVA, followed by Dunnet's test (***P≤ 0.001; ns = nonsignificant). (**F**) RLuc and FLuc activities were measured, and results are presented as RTA, relative to the activities obtained from the dl HIV-1 IRES vector transfected with the control DNA, set to 100%. The values are presented as the average (±SEM) from three independent experiments, with each performed in duplicate. Statistical analysis was performed using ANOVA, followed by Dunnet's test (ns, nonsignificant; * *P*≤ 0.05; ** *P*≤ 0.01; *** *P*≤ 0.001; **** *P*≤ 0.0001).

In cells, the biological functions of hnRNPK are regulated by post-translational modifications (PTMs) (54–56). Thus, we wondered if hnRNPK PTMs impacted the protein's ability to exert its ITAF function over the HIV-1 IRES. We selected and evaluated the impact of phosphorylations on residues serine (S) 216, S256, S284, S353 and tyrosine (Y) 458. We also included methylations on residues arginine (R) 256 and R299. PTM selection was based on reports associating the modifications with hnRNPK intracellular distribution, RNA affinity, and protein–protein interaction and their potential impact on mRNA translation (41,42,54,57–61). In the case of phosphorylation, S or Y residues were mutated to alanine (A) (non-phosphorylated) or aspartic acid (D) (phosphomimetic). For methylations, R residues were substituted by lysine (K). HEK293T cells were transfected with the dl HIV-1 IRES and the HA-hnRNPK or mutant HA-hnRNPK (mut-hnRNPK) plasmids. The overexpression of the HA-hnRNPK and mut-hnRNPK proteins, confirmed by western blotting, using an anti-hnRNPK (total protein) or an anti-HA antibody (recombinant) and GAPDH as a loading control (Figure 6A), did not affect cell viability (Figure 6B). Consistent with our previous results (Figures 4C and 4D), the overexpression of the HA-hnRNPK (wt) significantly increased HIV-1 IRES activity ($\sim$75% increase) over the control (−) (Figure 6C). When compared to HA-hnRNPK, none of the tested mutant proteins abrogated HIV-1 IRES activity (Figure 6C), however, the mut-hnRNPK differentially promoted, or not, the activity of the HIV-1 IRES. Stimulation of the HIV-1 IRES by the mutants over the control (−) was as follows: HA-mut-hnRNPK S216A ($\sim$91% increase) > S284A = S284/S353A (78% increase) > S284D = S353A = R256K ($\sim$58% increase) (Figure 6C). The HA-mut-hnRNPK S353D, S216D, S284/353D, Y458A, Y548D, R299K and R256/299K did not significantly stimulate HIV-1 IRES activity (Figure 6C).

Next, we focused on the hnRNPK mutants S216D and S284/353D, which lost the ability to stimulate HIV-1 IRES activity. Earlier reports showed that the S284/353D mutant is mainly cytoplasmic, while the S284/353A mutant is nuclear (41,62). Thus, the subcellular localization of S284/353D, S284/353A, S216A and S216D mutants was evaluated by IF in HEK293T cells. In agreement with previous reports (41,62), S284/353D was identified in the cytoplasm, while S284/353A remained mainly nuclear (Supplemental Figure S3A). IF results also show that the S216A mutant was mainly nuclear, while the S216D mutant was found in the cytoplasm (Supplemental Figure S3). This finding was further confirmed through a nuclear/cytoplasmic fractionation approach followed by western blot analysis using PARP as a marker for nuclear (N) extracts and GAPDH as a marker for cytoplasmic (C) extracts (Supplemental Figure S3B). Even though the molecular mechanism remains unclear (discussed below), our results support the notion that serine phosphorylation at S216 and S284/355 modulates hnRNPK's ability to promote HIV-1 IRES activity. Interestingly, we find that the nuclear localization of hnRNPK (found with mutants S216A and S284/353A) correlated with an increased HIV-1 IRES activity.

The phosphorylation of residue Y458, located within the nucleic acid-binding site of the KH3 domain, is expected to reduce, but not abolish, hnRNPK interaction with target mRNA (42,60). Unexpectedly, our results showed that Y458A exhibited effects similar to Y458D (42) on hnRNPK’s capacity to stimulate HIV-1 IRES activity. To further understand this apparent paradox, hnRNPK residue Y458 was mutated to S, glutamic acid (E), arginine (R), phenylalanine (F) or threonine (T). A previous report showed that hnRNPK mutant Y458F binds RNA to the same extent as the wild-type protein (42). HEK293T cells were transfected with the dl HIV-1 IRES and HA-hnRNPK or Y458 mut-hnRNPK plasmids. The overexpression of the HA-hnRNPK and Y458 mut-hnRNPK, as confirmed by western blot analyses, using an anti-hnRNPK (total protein) or an anti-HA antibody (recombinant) and GAPDH as a loading control (Figure 6D), did not affect cell viability (Figure 6E). As expected the HA-hnRNPK overexpression stimulated ($\sim$56% increase) HIV-1 IRES activity, while Y458A, Y458D, Y458E, Y458S yielded modest 18%, 10%, 5% and 6% increases, respectively (Figure 6F). Y458R ($\sim\;$11% decrease) induced a slight reduction in HIV-1 IRES activity. Mutants Y458F and Y458T enhanced HIV-1 IRES by 26% and 49%, respectively, but to a lower extent than wt-hnRNPK (Figure 6F). The results are discussed below.

### PRMT1-induced asymmetrical dimethylations impact HIV-1 gene expression and the activity of the HIV-1 IRES in HEK293T cells

Protein arginine methyltransferase 1 (PRMT1) is the only enzyme responsible for asymmetrically dimethylating hnRNPK on mainly five specific arginine residues (256, 258, 268, 296 and 299) (44). Since global PRMT1-induced arginine methylations do not impact the translational activity of cellular IRESs (63), nor do they affect various functions associated with hnRNPK, including RNA binding (44,55), we anticipated that PRMT1-induced methylations of hnRNPK would not alter the protein's stimulatory effect over the HIV-1 IRES activity. Contrary to our expectations, our results suggested that PRMT1-induced asymmetrical dimethylations of arginine residues (aDMAs) of hnRNPK are highly relevant for HIV-1 IRES activity (Figure 6C). If so, it was plausible to suspect that aDMAs of hnRNPK could also impact HIV-1 gene expression.

**Figure 7.**
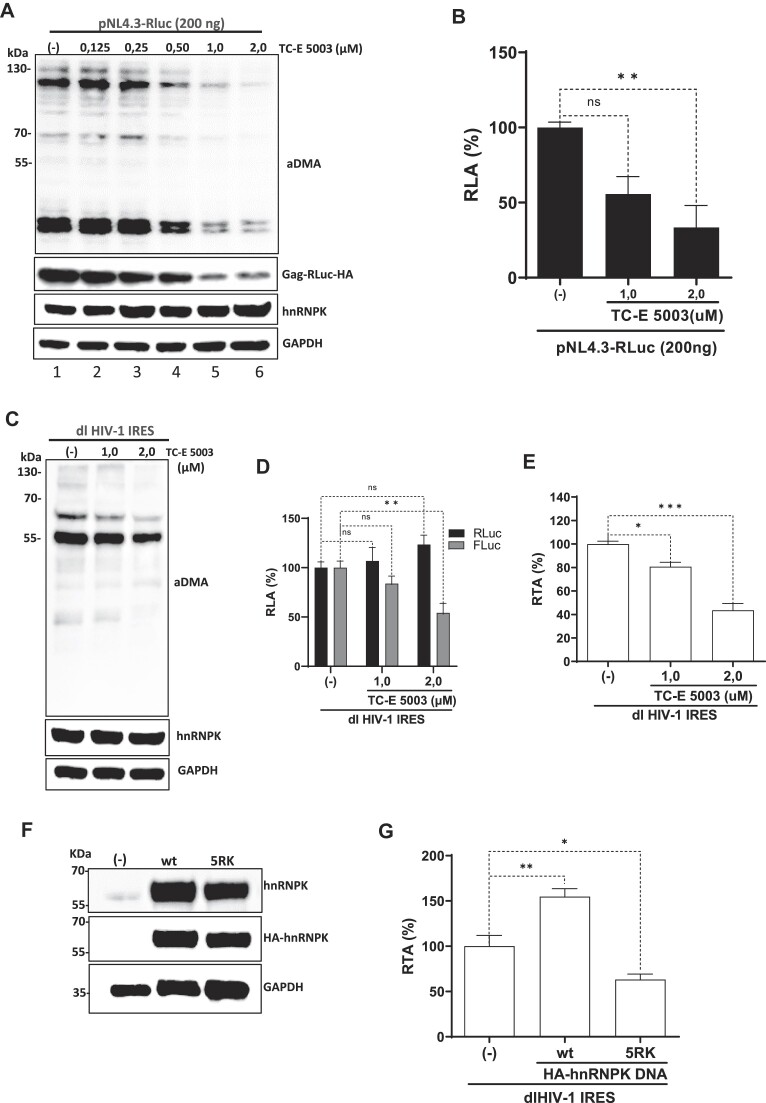
Inhibition of PRMT1 asymmetrical dimethylation impairs HIV-1 IRES activity. HEK 293T cells were cotransfected with the pNL-4.3-RLuc (200 ng) plasmid and 4 hpt treated, or not (only vehicle; (−)), with TC-E 5003 (0.125, 0.25, 0.50, 1 or 2 μM), a specific inhibitor of the PRMT1 activity. Total protein extracts were prepared 24 h post-treatment. (**A**) The PRMT1 activity (aDMA), and levels of the HIV-1 Gag-RLuc-HA fusion protein and endogenous hnRNPK in extracts from the untreated cell (lane 1) or from cells treated with at the different drug concentrations (0.125, 0.25, 0.50, 1 or 2 μM) were evaluated by western blot using GAPDH as a loading control. (**B**) RLuc activity was measured in proteins recovered from cells transfected with the pNL-4.3-RLuc plasmid and treated with 1 or 2 μM of TC-E-5003, and data are presented as RLA to the non-treated (−) cells set to 100%. Values shown are the mean (± SEM) from three independent experiments, with each performed in duplicate. Statistical analysis was performed using ANOVA, followed by Dunnet's test (** *P*≤ 0.01; ns, nonsignificant). (**C–E**) HEK293T cells were transfected with the dl HIV-1 IRES (200 ng) plasmid, and 4 hpt were treated, or not (only vehicle; (−)), with TC-E 5003 (1, or 2 μM). Total protein extracts were prepared 24 h post-treatment. (**C**) The PRMT1 activity (aDMA) and levels of endogenous hnRNPK in extracts from the untreated cell (lane 1) or from cells treated with 1 or 2 μM of TC-E 5003 were evaluated by western blot using GAPDH as a loading control. (**D, E**) RLuc and FLuc activities were measured, and data are presented as RLA (**D**) or RTA (**E**) relative to the non-treated (−) cells set to 100%. Values shown are the mean (± SEM) from six independent experiments, with each performed in duplicate. Statistical analysis was performed using ANOVA, followed by Dunnet's test (*P≤ 0.05; ** *P*≤ 0.01; *** *P*≤ 0.001; ns, nonsignificant). (**F**, **G**) An HA-hnRNPK mutant, 5RK, with the five arginine residues (256, 258, 268, 296 and 299) targeted by PRMT1 substituted by lysine was generated by site-directed mutagenesis of the HA-hnRNPK(wt) template plasmid (wt). HEK293T cells were cotransfected with the dl HIV-1 IRES (200 ng) together with HA-hnRNPK(wt) (200 ng) or the 5RK (200 ng) plasmid. Total protein extracts were prepared 24 hpt. (**F**) Western blots were performed to detect the expression level of total hnRNPK and HA-hnRNPK proteins using GAPDH as a loading control. (**G**) RLuc and FLuc activities were measured, and results are presented as RTA, relative to extracts obtained from cells transfected with the dl HIV-1 IRES plasmid together with an irrelevant DNA (pSP64-poly(A)) set to 100%. Values shown are the mean (± SEM) from five independent experiments, with each performed in duplicate. Statistical analysis was performed using ANOVA, followed by Dunnet's test (* *P*≤ 0.05; ** *P*≤ 0.01; *** *P*≤ 0.001).

In our effort to establish if PRMT1 regulates HIV-1 protein synthesis, the impact of *N*,*N*′-(Sulfonyldi-4,1-phenylene)bis(2-chloroacetamide) (TC-E 5003) (64), a selective PRMT1 inhibitor (IC_50_ = 1.5 μM) on HIV-1 gene expression was evaluated. For this, HEK293T cells were transfected with pNL4.3-RLuc (200 ng) and treated or not (vehicle alone) with TC-E 5003 (0.125-2 μM). The treatment of HEK293T cells with low TC-E 5003 concentrations between 0.125-2 μM did not impact cell viability (Supplemental Figure S2C), but higher concentrations of TC-E 5003 (4-8 μM) significantly reduced cell viability (Supplemental Figure S2B). Endogenous aDMA levels of cellular proteins were monitored by western blot analyses using a commercial mix of antibodies that recognizes endogenous aDMA (Figure 7A). The treatment of cells with TC-E-5003 (0.125–2 μM) markedly reduced endogenous aDMA signals at drug concentrations of (0.5, 1 and 2 μM; Figure 7A, lanes 4, 5 and 6) without an evident impact on endogenous GAPDH control or the abundance of endogenous hnRNPK (Figure 7A). Western blot analysis using an anti-HA antibody revealed a sharp reduction of Gag-RLuc-HA protein in cells treated with 1 and 2 μM TC-E-5003 (Figure 7A, lanes 5 and 6). Consistent with this observation RLuc activity was also significantly reduced in cells treated with 1 μM ($\sim$46% reduction) or 2 μM ($\sim$68% reduction) of TC-E 5003 (Figure 7B). These observations strongly suggest that PRMT1-induced aDMA plays a role in HIV-1 gene expression.

Next, we directly assessed the effect of the selective PRMT1 inhibitor on HIV-1 IRES activity. For this, HEK293T cells were transfected with the dl HIV-1 IRES plasmid (200 ng) and treated, or not (vehicle alone), with TC-E 5003 (0.125–2 μM). Global protein aDMA was followed by western blot analyses (Figure 7C). As shown earlier, the treatment of cells with TC-E 5003 (1 or 2 μM) reduced aDMA, with no apparent impact on hnRNPK or GAPDH abundance (Figure 7C). RLuc activity in TC-E 5003 (1 or 2 μM) treated cells showed an increasing trend ($\sim$23% increase at 2 μM of the drug) (Figure 7D). In contrast, the FLuc activity was significantly decreased ($\sim$46% reduction) at the highest concentrations of TC-E-5003 (2 μM) (Figure 7D). Results are also expressed as RTA to illustrate better the significantly lower activity ($\sim$55% reduction) of the HIV-1 IRES in cells treated with TC-E 5003 (2 μM) (Figure 7E). As an additional control, cell extracts obtained from HEK293T expressing RLuc and FLuc from the dl HIV-1 IRES plasmid were directly mixed, or not, with TC-E 5003 (2 μM). Results show that the PRMT1 inhibitor does not affect RLuc or FLuc enzymatic activity (Supplemental Figure S4).

The effect of TC-E 5003 was likely pleiotropic because PRMT1 interacts with several proteins relevant to HIV-1 IRES activity, such as eIF5A, Stau1, and hnRNPA1 (65). In addition, PRMT1 methylates hnRNPA1 (66). To directly address whether PRMT1-induced aDMAs of hnRNPK impacted the protein's ITAF function over the HIV-1 IRES, an HA-tagged hnRNP K-5RK mutant (5RK) was constructed. In this mutant, the five arginine residues (256, 258, 268, 296,and 299) targeted by PRMT1 were substituted by lysine. HEK293T cells were transfected with the dl HIV-1 IRES DNA and the plasmids expressing HA-hnRNPK or the HA-5RK mutant. The overexpression of the 5RK, confirmed by western blot analyses (Figure 7F), did not impact cell viability (Supplemental Figure S2C). The overexpressed 5RK remained predominantly, but not exclusively, nuclear (Supplemental Figure S3A). Luciferase activities were monitored, and results presented as RTA show that the overexpression of HA-hnRNPK(wt) increased HIV-1 IRES activity, while the overexpression of 5RK reduced HIV-1 IRES activity ($\sim$35%) (Figure 7G).

Next, we decided to exclude the possibility that the observed effect was due to the HA-tag or selected amino acid substitutions used to mutate hnRNPK. Experiments were therefore conducted in HEK293T cells using the previously characterized GFP-hnRNPK and GFP-5RG mutant, in which the arginine residues 256, 258, 268, 296 and 299 of hnRNPK were substituted by glycine (44). In contrast to the GFP-hnRNPK, the expression of the GFP-5RG mutant protein (Supplemental Figure S5A) non-significantly reduced HIV-1 IRES activity ($\sim$25%) in HEK293T cells (Supplemental Figure S5B), confirming that hnRNPK requires the PRMT1-induced aDMAs to stimulate HIV-1 IRES activity.

We next sought to confirm that the HIV-1 IRES stimulation by hnRNPK overexpression was also observed in HeLa cells. For this, HeLa cells were transfected with dl HIV-1 IRES or dl HIV-1 1-104 (lacking IRES activity) plasmid alone or in combination with plasmids encoding for the HA-hnRNPK or the HA-5RK proteins. The expression of HA-hnRNPK, the HA-5RK, FLuc, and RLuc, was confirmed by western blot using GAPDH as a loading control (Supplemental Figure S6A). As a negative control, non-transfected cells (NT) extracts were also loaded in the western blot assay (Supplemental Figure S6A, lane 7). As expected, RLuc was detected when cells were transfected with either the dl HIV-1 1-104 (Supplemental Figure S6A, lanes 1–3) or the dl HIV-1 IRES (Supplemental Figure S6A, lanes 4–6), while FLuc was detected only when cells were transfected with the dl HIV-1 IRES plasmid (Supplemental Figure S6A, lanes 4–6). Co-transfection of HA-hnRNPK, but not HA-5RK, increased the apparent amount of FLuc without impacting the amount of RLuc (Supplemental Figure S6A, lanes 5–6). To further validate our observations, HeLa cells were transfected with the dl HIV-1 IRES plasmid with an irrelevant DNA (−) or together with plasmids encoding for HA-hnRNPK or the HA-5RK. The expression of the recombinant proteins was confirmed by IF (Supplemental Figure S6B). As anticipated, both the cap-dependent RLuc, and the HIV-1 IRES-dependent FLuc proteins could be readily detected by IF in HeLa cells transfected with the dl HIV-1 IRES plasmid (Supplemental Figure S6B). The mean fluorescence intensity (MFI) values for RLuc and FLuc obtained from the imaging data were used to calculate the RTA as previously described (22). The RTA value obtained in the absence of a recombinant protein (−) was set to 100%. Consistent with our previous observations, the overexpression of HA-hnRNPK, but not HA-5RK, enhanced HIV-1 IRES activity in HeLa cells (Supplemental Figure S6C). In conclusion, PRMT1-induced aDMAs enable hnRNPK to stimulate HIV-1 IRES activity.

### HnRNPK and the 5RK are equivalently associated with the HIV-1 vRNA but interact differently with known HIV-1 IRES ITAFs

To gain insight into the possible mechanism by which hnRNPK and 5RK exert their function over HIV-1 vRNA translation, HeLa cells were cotransfected with the HA-hnRNPK or HA-5RK expression plasmids, and the pNL4.3 DNA. Viral replication was monitored by combined IF and fluorescence *in situ* hybridization (IF/*FISH*) for p24 and the vRNA (Supplemental Figure S7A) (67). The co-localization between p24 (Supplemental Figure S7B) or the recombinant hnRNPK proteins and the vRNA (Supplemental Figure S7C) was determined by calculating the Mander's coefficient per frame and expressed as a percentage. Gag (p24) was equivalently co-localized with the vRNA either in the presence or not of HA-hnRNPK or the HA-5RK mutant (Supplemental Figure S7B). In agreement with previous findings showing that PRMT1-induced arginine methylations of hnRNPK did not impact the ability of the protein to bind its target RNA (44,55), both HA-hnRNPK and HA-5RK equivalently co-localized with the HIV-1 vRNA (Supplemental Figure S7C).

**Figure 8.**
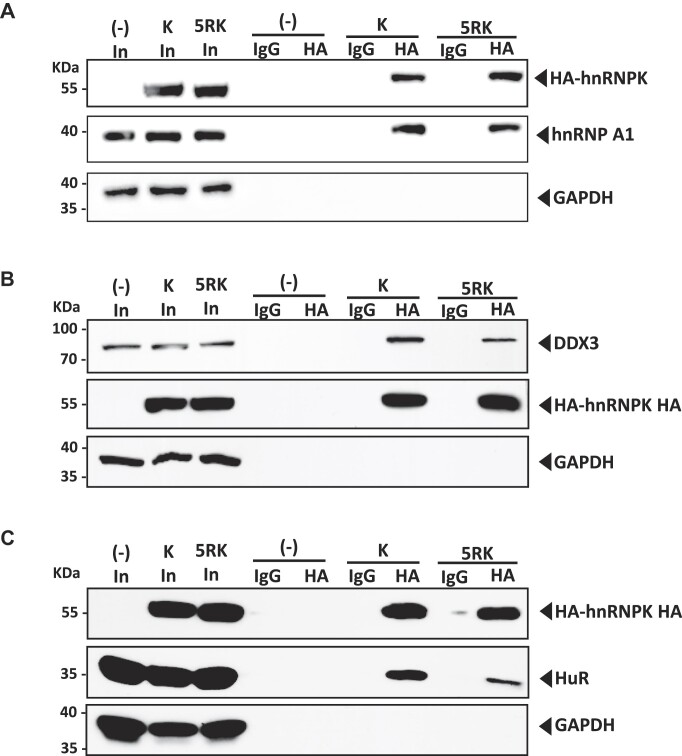
HnRNPK and 5RK differentially interact with other known ITAFs for the HIV-1 IRES. HEK293T cells were cotransfected with the dl HIV-1 IRES DNA and the HA-hnRNPK or the HA-5RK encoding plasmids. 48 h later, cells were lysed, and immunoprecipitation (IP) assays were performed using protein A/G agarose coated with IgG or the anti-HA antibody. The beads were washed extensively and incubated with loading buffer at 95ºC, and the supernatant from whole-cell lysate (input; In) of each sample and IP fractions (anti-IgG or anti-HA) were used for western blotting. Western blotting was performed using (**A**) anti-HA, anti-hnRNPA1, and anti-GAPDH antibodies, (**B**) anti-DDX3, anti-HA and anti-GAPDH antibodies, or (**C**) anti-HA, anti-HuR, and anti-GAPDH antibodies. Horseradish peroxidase (HRP)-conjugated protein A/G was used to detect the primary antibodies.

In cells, hnRNPK interacts with several known ITAFs for the HIV-1 IRES, including hnRNPA1, DDX3, and HuR (33,34). Asymmetric arginine demethylation of hnRNPK by PRMT-1 impacts the ability of hnRNPK to interact with protein partners such as DDX-3, c-Src and p53 (44,68–70). Therefore, we wondered whether hnRNPK and the 5RK mutant equivalently interacted with known HIV-1 IRES ITAFs, hnRNPA1, DDX3 and HuR (Figure 8). For this, HEK293T cells were transfected with the dl HIV-1 IRES and the expression plasmid for HA-hnRNPK or HA-5RK, and 48 hpt, co-immunoprecipitation (CoIP) assay were performed using protein G-agarose (PGA)-beads loaded with either an anti-HA, or anti-IgG control antibody coupled with a western blot analysis. Proteins were detected in whole cell extracts (input; In) from cells transfected with the respective expressing plasmid (Figure 8). As a loading control, GAPDH was detected using an anti-GAPDH-specific antibody. A comparison of GAPDH in the input suggests that similar amounts of protein were loaded (Figure 8). Capture and IP of the HA-hnRNPK and HA-5RK were confirmed by western blotting using an anti-HA antibody (Figure 8). HA-hnRNPK and HA-5RK could be detected when the IP assay was performed using PGA beads coated with the anti-HA, but not when beads were covered with the anti-IgG control antibody (Figure 8). In the presence of the dl HIV-1 IRES vector, endogenous hnRNPA1 (Figure 8A), DDX3 (Figure 8B), and HuR (Figure 8C) co-immunoprecipitated with HA-hnRNPK and HA-5RK. However, compared to HA-hnRNPK, DDX3 and HuR were poorly enriched when HA-5RK was used (Figure 8).

### HnRNPK is an ITAF for the HTLV-1 IRESs, but not for the sHBZ IRES

**Figure 9.**
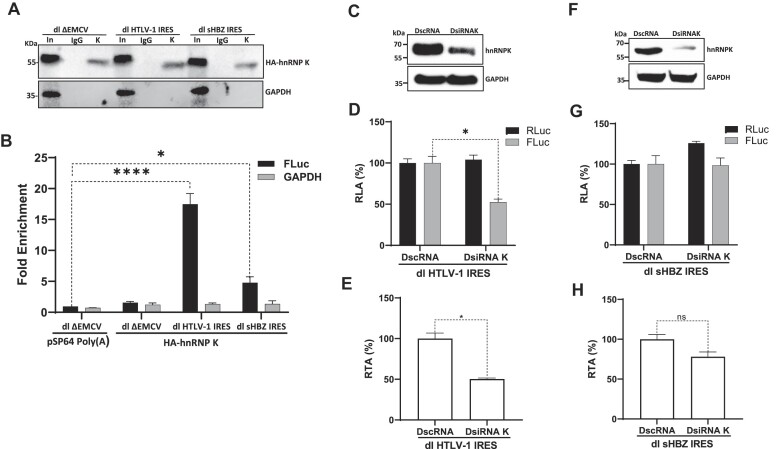
Reduction of endogenous hnRNPK negatively impacts HTLV-1 IRES without affecting sHBZ IRES activity. HEK 293T cells were transfected with the dl ΔEMCV, dl HTLV-1 IRES, or dl sHBZ IRES plasmids, proteins and RNAs were ultraviolet (UV) cross-linked (254 nm-irradiated with 400 mJ/cm^2^). Endogenous hnRNPK was immunoprecipitated (IP) from cell extracts using protein A/G-agarose (PGA)-beads loaded with an anti-hnRNPK antibody or using PGA-beads loaded with an anti-IgG antibody as a negative control. (**A**) The hnRNPK protein present in the input extracts and the IPs were evaluated by western blotting using an anti-HA antibody. As a loading control, GAPDH was detected using an anti-GAPDH-specific antibody. The protein A/G HRP conjugated was used as the secondary antibody. (**B**) The quantity of FLuc encoding RNA (FLuc) or GAPDH encoding RNA (GAPDH) covalently bound to hnRNPK was determined by an RT-qPCR assay as described in (46). RNA fold enrichment, as defined in (46), obtained in the IP from cells transfected with the dl ΔEMCV DNA, was set to 1. Values shown are the mean (± SEM) from two independent experiments, with each RT-qPCR assay performed in duplicate. Statistical analyses were performed using the ANOVA Kruskal-Wallis test (*P*< 0.05). (**C-H**) HEK293T cells were cotransfected with (**C–E**) the dl HTLV-1 IRES (200 ng), or (**F–H**) the dl sHBZ IRES (200 ng) plasmids, and a DscRNA (10 nM) control or DsiRNAK RNAs. Reduction of endogenous hnRNPK was monitored by western blot using GAPDH as a loading control (**C** and **F**). RLuc and FLuc activities were measured at 24 hpt, and data are presented as RLA (**D** and **G**) or as RTA (**E** and **H**). The RLA and RTA values obtained in the absence of when using the DscRNA were set to 100%. Values represent the mean (± SEM) from three independent experiments, with each conducted in duplicate. Statistical analysis was performed by a t-student test (ns, nonsignificant; *P < 0.05).

Next, we questioned whether hnRNPK could also modulate the activity of other retroviral IRESs. To address this issue, we selected to assess the HTLV-1 IRES, present within the 5′UTR of the human T cell lymphotropic virus-type 1 (HTLV-1) vRNA (37), and the sHBZ IRES, found within the 5′UTR of the spliced version of the antisense HTLV-1 mRNA encoding for the HTLV-1 basic leucine zipper protein (sHBZ) (38). First, we sought to determine if hnRNPK interacts with the 5′leader of the HTLV-1 vRNA or the antisense mRNA encoding sHBZ *in vivo*. HEK293T cells were transfected with dl ΔEMCV, dl HTLV-1 IRES or dl sHBZ IRES plasmids with the HA-hnRNPK expressing plasmids. HEK293T cells were transfected with dl ΔEMCV and the pSP64 Poly(A) plasmid as a control. Cells were treated with ultraviolet (UV) light to covalently cross-linking (CL) proteins to RNA (46). Total proteins were recovered, and HA-hnRNPK was IP using PGA-beads loaded with an anti-HA antibody. HA-hnRNP K was detected in the input from transfected cells (Figure 9A). As a loading control, GAPDH was detected using an anti-GAPDH-specific antibody. A comparison of GAPDH in the whole cell extracts or input (In) suggests that similar amounts of protein were loaded (Figure 9A). Capture and IP of the recombinant protein was confirmed by western blotting using an anti-HA (Figure 9A). HA-hnRNPK could be detected when the IP assay was performed using PGA beads coated with the anti-HA antibody but not when anti-IgG coated PGA beads were used (Figure 9A). After the IP, proteins were removed by proteinase K treatment, and FLuc RNA and GAPDH RNA were quantified by real-time RT-qPCR (2,46). FLuc RNA and GAPDH RNA were also quantified in the total input. The fold enrichment (FE) of FLuc RNA and GAPDH RNA in the IP from cells transfected with the dl ΔEMCV in combination with the pSP64 Poly(A) was set to 1 (unspecific pulldown/binding). FE shows the enrichment of FLuc RNA or GAPDH RNA found in the IP using the anti-HA antibody (specific IP) over the FLuc RNA or GADPH RNA found in the IP using the anti-IgG control antibody (unspecific IP). The FLuc RNA or GAPDH RNA from each IP was normalized to the FLuc RNA or GAPDH RNA found in the input to account for any differences in the RNA sample preparation (46). Results showed that dl HTLV-1 IRES (FE $\sim$17) and dl sHBZ IRES (FE $\sim$5) RNAs were significantly enriched in the IPs using anti-HA antibody over IPs with the anti-IgG control antibody, while GAPDH RNA was not (Figure 9B). Based on the UV-CLIP/RT-qPCR data, we conclude that hnRNPK interacts with the 5′leader of the HTLV-1 vRNA more efficiently than with the *shbz* mRNA in cells.

Next, we evaluated the impact of endogenous hnRNPK knockdown on the activity of the HTLV-1 IRES and sHBZ IRES by transfecting HEK293T with the dl HTLV-1 IRES (Figure 9C) or dl sHBZ IRES (Figure 9D) together with the DsiRNAK (10nM) or the DscRNA (10 nM) control RNA. Targeting the endogenous hnRNPK (Figure 9C) reduced FLuc activity from the dl HTLV-1 IRES ($\sim$48% decrease) without significantly impacting on RLuc activity (Figure 9D). This observation is better supported when the data are presented as RTA where the HTLV-1 IRES ($\sim$50% reduction) activity decreased in cells treated with the DscRNAK RNA (Figure 9E). Interestingly, the decrease of endogenous hnRNPK levels (Figure 9F), did not impact the activity of the sHBZ IRES (Figure 9G and H).

**Figure 10.**
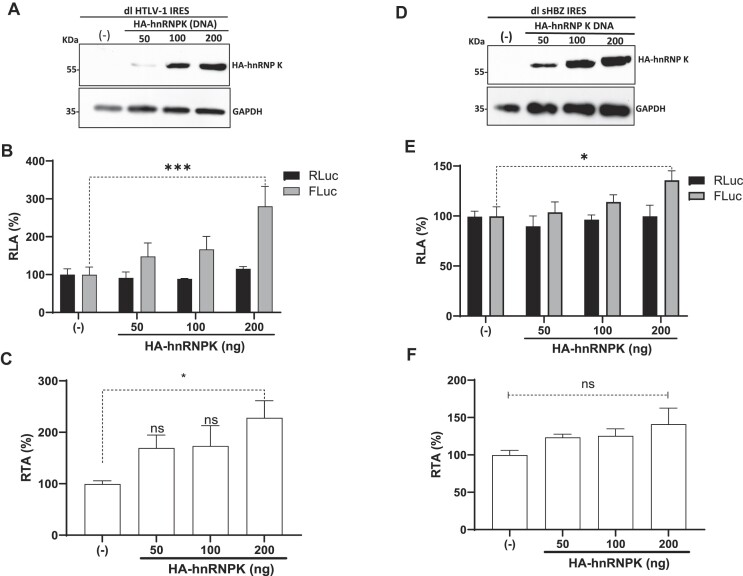
Overexpression of hnRNPK promotes the activity of the HTLV-1 IRES but not from the sHBZ IRES. HEK293T cells were cotransfected with (**A–C**) the dl HTLV-1 IRES (200 ng), or (**D–F**) or the dl sHBZ IRES (200 ng) plasmids, and the HA-hnRNPK encoding plasmid. The presence of the overexpressed HA-hnRNPK protein was confirmed by western blot using GAPDH as a loading control (**A** and **D**). RLuc and FLuc activities were measured at 24 hpt, and data are presented as RLA (**B** and **E**) or RTA (**C** and **F**). The RLA and RTA values obtained in the absence of HA-hnRNPK plasmid were set to 100%. Values represent the mean (± SEM) from three independent experiments, each conducted in duplicate. Statistical analysis was performed by ANOVA (*P< 0.05).

To determine whether the overexpression of HA-hnRNPK, which does not impact plasmids cryptic promoter activity or induces alternative splicing of the dl-RNAs (Supplemental Figure S8), affected HTLV-1 and sHBZ IRES activity, the dl HTLV-1 IRES (Figure 10A–C) or dl sHBZ IRES (Figure 10D–F) and the HA-hnRNPK (50, 100, 200 ng) expression plasmids or an irrelevant DNA (−) were co-transfected into HEK293T cells. The expression of the HA-hnRNPK protein was confirmed by western blot (Figure 10A and D). Luciferase activities were measured, and data were expressed as RLA (Figure 10B and E) or RTA (Figure 10C and F). The overexpression of HA-hnRNPK did not alter RLuc activity in any of the bicistronic mRNAs (Figure 10B and E), but it stimulated the activity from the HTLV-1 IRES ($\sim$280% increase; 200 ng DNA) (Figure 10B). The impact of hnRNPK over the HTLV-1 IRES is better appreciated when the results are presented as RTA (Figure 10C). The overexpression of HA-hnRNPK had no significant effect over the sHBZ IRES (Figure 10E and F). These observations suggest that in HEK293T cells, hnRNPK also acts as an ITAF for the HTLV-1 IRES without impacting the activity of the sHBZ IRES.

## Discussion

In this study, we identify hnRNPK, a member of the nuclear-enriched poly (C)-binding protein (PCBP) family, as a *bona fide* ITAF that stimulates the activity of the HIV-1 (Figure 1–4) and HTLV-1 IRESs (Figures 9 and 10). HnRNPK depletion reduces, while its overexpression stimulates activity from the HIV-1 and HTLV-1 IRESs (Figures 3, 9 and 10). Interestingly, when compared to the 5′UTR of the HTLV-1 mRNA, hnRNPK does not efficiently bind the 5′UTR of the sHBZ mRNA, nor does it act as an ITAF for the sHBZ IRES (Figures 9 and 10), suggesting that not all retroviral IRES require the same subset of ITAFs for function. These findings were not unexpected as other ITAFs for retroviral IRESs are also virus-specific. For example, the polypyrimidine-tract-binding protein (PTB) is needed for the activity of the IRESs present within the Moloney murine leukemia virus and mouse mammary tumor virus vRNAs (46,71), but it does not have a function in translation initiation mediated by the HIV-1 IRES (25,46).

PTMs regulate the biological functions of hnRNPK (54–56). In agreement, results from a basic screening approach showed that the phosphorylation in S216, S284, S353 and PRMT-1 induced asymmetrical dimethylation of hnRNPK impacted the ability of the protein to stimulate HIV-1 IRES activity. As previously reported (41,62), the S284/S353D hnRNPK mutant accumulated in the cell cytoplasm (Supplemental Figure S3). Strikingly, when S284/S353D hnRNPK was overexpressed the stimulation of HIV-1 IRES activity was reduced (Figure 6). In contrast, the S284/S353A hnRNPK mutant, which is mainly nuclear (Supplemental Figure S3 and (62)), enhanced HIV-1 IRES activity (Figure 6). In cells, mitogen-activated protein kinase/extracellular-signal-regulated kinase (MAPK/ERK) phosphorylates hnRNPK at S284 and S353 (41). Interestingly, hnRNPK translation-regulatory activity dependent on phosphorylation of hnRNPK on S284/353 by MAPK/ERK is not exclusively observed for the HIV-1 IRES (Figure 6), as IRES-mediated translation initiation of the *c-myc* mRNA is also increased when the wild-type and S284/353A hnRNPK are overexpressed (30,72). In contrast, when phosphorylated at S284/353, hnRNPK inhibits translation of the Lox mRNA (41). HnRNPK regulates gene expression by integrating the cross-talk between kinases and hnRNPK-interacting partners (56). Therefore, it is not surprising that PTMs, such as phosphorylation at S284/353 in hnRNPK, differentially impact target mRNAs. This would most likely occur through different mechanisms, presenting hnRNPK as a central node for a complex translational control network in normal physiology and during virus replication.

Regarding the amino acid in position 458 (Figure 6), a previous report showed that the substitution of amino acid 458 did not alter the structure of hnRNPK (42). This substitution, however, modified the chemical environment of the C-terminal end of the protein, likely changing long-range interactions of the tyrosine side chains with the core KH domain, impacting the protein's ability to interact with its target RNA (42). Although highly speculative, it is plausible that threonine could partially restore the interaction of site 458 with the core KH domain required to enable hnRNPK to function as an ITAF for the HIV-1 IRES activity. Based on our results (Figure 6), the impact of Y458 phosphorylation on HIV-1 IRES activity remains inconclusive as the chosen experimental approach could not adequately assess it. Further experiments will, therefore, be necessary to fully understand the molecular mechanism associated with the impact of residue 458 on the ability of hnRNPK to stimulate HIV-1 IRES activity.

We included R256K, R299K and R256/299K mutants when designing our experiments, because residue R256 participates in hnRNPK interaction with the ribosomal protein 19 (RPS19) (68), and R299 is the dominant site of PRMT1 methylation (73). Our results suggest that PRMT-1 does not impact HIV-1 IRES-mediated translation initiation by interfering with hnRNPK-40S ribosomal interaction (through RPS19) as mutant R256K stimulated HIV-1 IRES activity (Figure 6C). What was unexpected was that R299K and R256/299K mutants were unable to stimulate HIV-1 IRES activity (Figure 6C), suggesting that hnRNPK PRMT1-induced aDMAs are required to stimulate the activity of the HIV-1 IRES. This observation was confirmed by pharmacological inhibition of PRMT1 activity and using the 5RK mutant (Figure 7 and Supplemental Figures S5 and S6). This finding was unexpected since an earlier report showed that the pharmacological inhibition of PRMT1 had no observable impact on the translational activity of the IRESs found in mRNAs encoding Cyclin D1 (CCD1), cellular Myelocytomatosis (c-MYC), hypoxia-inducible factor-1α (HIF1α), estrogen receptor α (ESR1), and cyclin-dependent kinase inhibitors 1B (CDKN1B) (63). The mechanism by which PRMT1 methylated hnRNPK affects HIV-1 IRES activity remains unclear. However, in agreement with reports indicating that PRMT1-induced aDMAs of hnRNPK do not influence the protein's ability to bind RNA (44,55), we observed that in HIV-1 replicating cells and overexpressing the wt-hnRNPK or the 5RK mutant, both recombinant proteins co-localize with the vRNA equivalently (Supplemental Figure S7). A possible clue to understanding our results emerges from reports indicating that PRMT1-induced aDMAs of hnRNPK affect the ability of the protein to interact with some of its molecular partner's regulating in this way its biological function (44,68). In agreement with these reports, we find that in the presence of the dl HIV-1 IRES RNA, the wt-hnRNPK and 5RK mutant differentially interact with DDX3 and HuR, two known HIV-1 IRES ITAFs (Figure 8). Interestingly, and entirely consistent with an earlier report (68), showing that both DDX3 and HuR are enriched when IPs are conducted using a methylated version of hnRNPK, we find when using the HA-5RK mutant DDX3 and HuR are less enriched (Figure 8). A potential role of the hnRNPK-DDX3 interaction in translational control has not been reported. However, the hnRNPK-HuR complex, evidenced only in proliferating cells (33), regulates translation of the p21 mRNA (74). Whether the decrease in 5RK interaction with DDX3 or HuR observed by co-immunoprecipitation is solely responsible for the reduced ability of the hnRNPK mutant to stimulate HIV-1 IRES remains inconclusive. Nonetheless, our results do highlight the relevance of the ribonucleoprotein complex that assembles on the HIV-1 vRNA to enable fine-tuning of IRES activity.

The results obtained with the hnRNPK mutants suggest a model that links nuclear events with the rate of cap-independent translation of the HIV-1 vRNA. Noteworthy is that hnRNPA1 associates with the vRNA in the nucleus and is exported with the vRNA as part of an RNP to modulate HIV-1 IRES-mediated translation initiation (11). Thus, it is tempting to speculate that aDMAs of hnRNPK mediated by PRMT1 enhance specific nuclear protein–RNA and protein-protein interactions, favoring the assembly of an efficient translational RNP-complex required later to promote HIV-1 IRES activity (1). In support of this possibility is the model proposed for cellular IRESs that suggests the nuclear assembly and transit to the cytoplasm of translation-competent RNP complexes (2,30,75,76). An alternative possibility that cannot be discarded is that the amount of endogenous hnRNPK found in the cytoplasm is sufficient to ensure HIV-1 IRES-mediated translation initiation. This last model is supported by the observation that HIV-1 gene expression does not induce a redistribution of hnRNPK to the cytoplasm (Figure 2,Supplemental Figure S1). Adding further complexity to its role in HIV-1 IRES-mediated translation initiation are several reports that show that endogenous hnRNPK actively modulates the translation of cellular and viral mRNAs by using different molecular mechanisms. For some mRNAs, hnRNPK binds to their 3′UTR, impeding 80S ribosome assembly. For others, hnRNPK binds to the 5′UTR, altering RNA structure or affecting the recruitment of other proteins involved in translation initiation (30,31,43,72,77–81). So, the precise molecular mechanism hnRNPK uses to promote HIV-1 IRES activity is difficult to anticipate without further work. However, we suspect that the action of hnRNPK on HIV-1 IRES activity is linked with its ability to interact with other partner proteins (Figure 8). Further experiments will be required to determine how hnRNPK interacting partners impact HIV-1 IRES activity.

In cells, hnRNPK is a component of several dynamic complexes whose composition changes in response to their location and the cellular environment (33). Biological functions ascribed to hnRNPK appear to depend on specific complexes in which hnRNPK is found. For example, in the context of HIV-1, as part of the Nef-associated kinase complex, hnRNPK increases vRNA transcription (26), and when hnRNPK interacts with Rev, it leads to the enhancement of Rev-mediated RRE-dependent gene expression (28,29). HnRNPK has more than 100 identified protein partners, some known to play a role in translational control (33). In this regard, our findings open new avenues of research as several other proteins, known to interact with hnRNPK to modulate translation initiation of its substrate mRNAs (68), may also be relevant for HIV-1 IRES activity.

In conclusion, we provide evidence that hnRNPK acts as a genuine ITAF for the HIV-1 and HTLV-1 IRESs, promoting their activity and identifying a novel and additional function of this multifunctional RBP in retroviral gene expression.

## Supplementary Material

gkad1221_Supplemental_File

## Data Availability

All data are available upon request.
