# Supplementary material for: Heterogeneous nuclear ribonucleoprotein K promotes cap-independent translation initiation of retroviral mRNAs
Source: Nucleic Acids Res. 2024 Jan 2;52(5):2625–47. doi: 10.1093/nar/gkad1221 (PMC10954487; doi:10.1093/nar/gkad1221)
Supplement: gkad1221_Supplemental_File [file gkad1221_supplemental_file.docx]

**Supplemental Figures:**

**"H**eterogeneous nuclear ribonucleoprotein K **promotes cap-independent translation initiation of retroviral mRNAs."**

**Fuentes, Olguín, et al. 2023. [https://doi.org/10.1093/nar/gkad1221]**

**
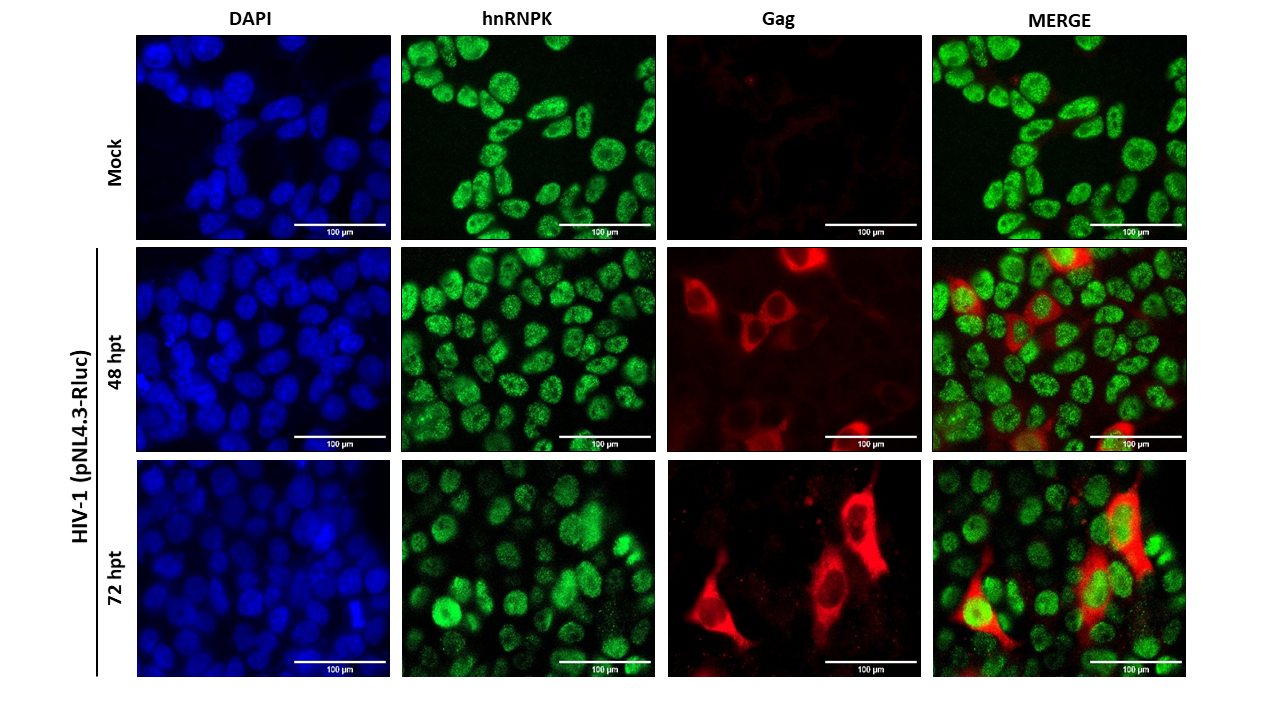
**

**Figure S1:** **Localization of endogenous hnRNP K in HEK293T cells expressing the NL4.3-RLuc proviral clone.** HEK293T cells were transfected with 200 ng of pCMV3-SP-PolyA or pCMV3-pNL4.3-RLuc-HA plasmids. At 48 and 72 hpt, cells were fixed (4 % PFA) and permeabilized (PBS-Triton) as indicated in the Material and Method section, and protein localization was evaluated by indirect immunofluorescence (IF) using a rabbit anti-p24, and a mouse anti-hnRNP K as primary antibodies. As secondary antibodies, an Alexa 647 donkey anti-rabbit (red) and Alexa 488 donkey anti-mouse (green) were used. 4',6-diamidino-2-phenylindole (DAPI), was used to stain the nucleus (blue). The images were captured with 63X magnification using a ZEISS microscope, axio observer D.1 model, and processed with the ImageJ program. Scale bar = 100 µm

**
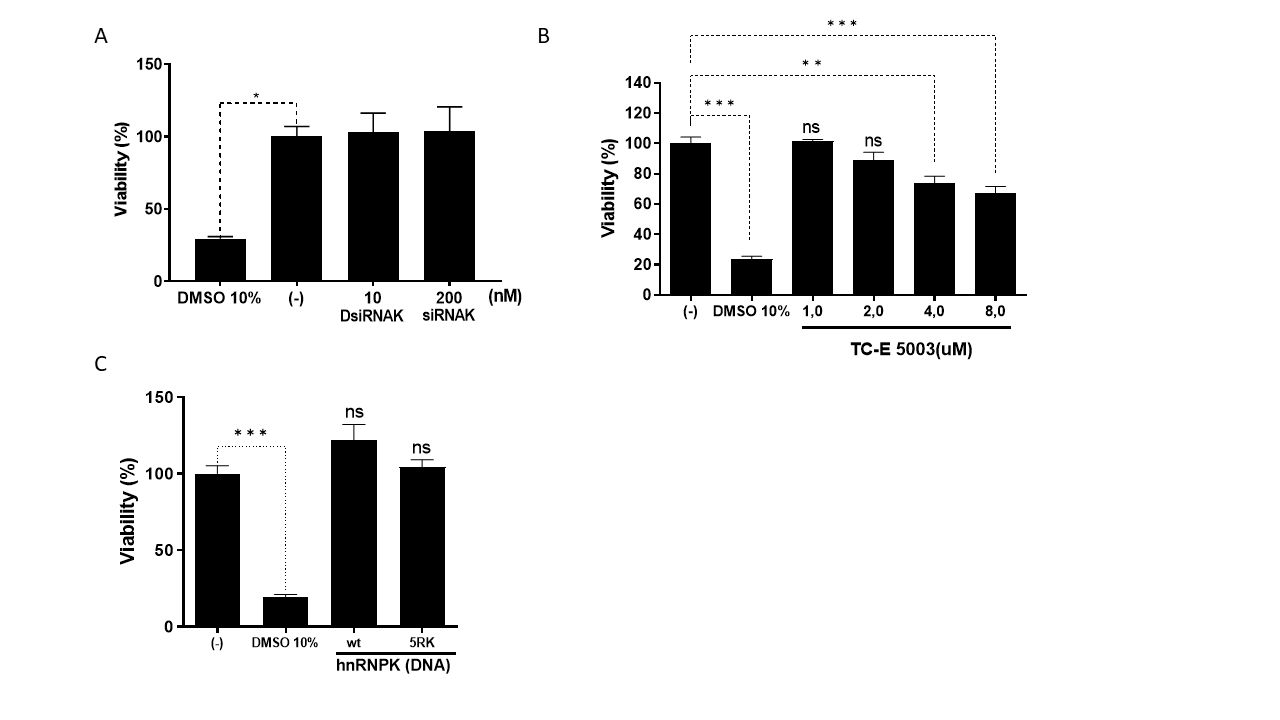
**

**Figure S2:** **Viability assay.** The viability of HEK293T cells was determined as described in the Materials and Method section using dimethylsulfoxide (DMSO, 10%) as a control for cell death. **(A)** Cells were transfected with DsiRNA K at 10 nM or siRNA K at 200 nM. Data are expressed in (%) relative to the viability of cells transfected with DscRNA, set to 100%. **(B)** Cells were treated with increasing concentrations of inhibitor TC-E 5003 at 1,0- 8,0 μM for 24 h. Data are expressed in (%) relative to the viability of cells in the presence of the drug vehicle (-) used as a negative control, set to 100%. **(C)** HEK293T cells were transfected with pCMV3-HA-hnRNPK (wt) or pCMV3-HA-hnRNPK 5RK plasmid. Data are expressed in (%) relative to the viability of cells transfected with pSP64 PolyA DNA set to 100%. Values shown are the mean (+/- SEM) from three independent experiments, with each performed in duplicate. Statistical analysis was performed using ANOVA, followed by Dunnet's test (*=P≤0.05; ** =P≤0.01; ***=P≤0.001; ****=P≤0.0001; ns= nonsignificant).

**
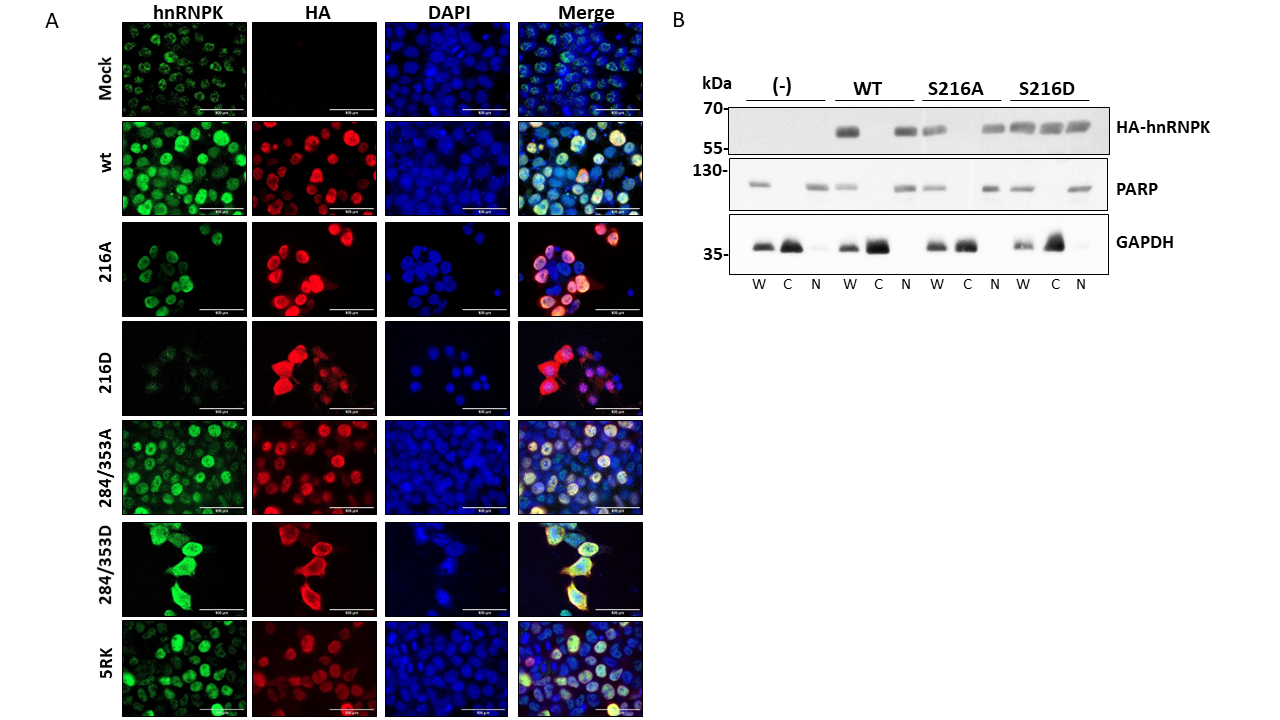
**

**Figure S3:** **Detection of HA-hnRNPK and mutant proteins in HEK293T cells.** **(A)** HEK293T cells were cotransfected with 200 ng of dl HIV-1 IRES plasmid, and with 200 ng of the pCMV3-SP-PolyA or pCMV3-HA-hnRNPK (wt) or mutants vector*s*, 24 hpt were fixed (PFA 4%) and permeabilized (PBS-Triton). Cells were washed and incubated with the primary antibodies rabbit anti-HA and mouse anti-hnRNPK. An Alexa 647 donkey anti-rabbit (red) and Alexa 488 donkey anti-mouse (green) were used as secondary antibodies. 4',6-diamidino-2-phenylindole (DAPI) was used to stain the nucleus (blue). The images were captured with 63X magnification using a ZEISS microscope, axio observer D.1 model, and processed with the ImageJ program. Scale bar = 100 µm **(B)** Western blot analysis of whole cell lysates (W), nuclear (N), and cytoplasmic (C) extracts obtained from HEK293T cells transfected with pSP64-poly(A), pCMV3-HA-hnRNPK wt or pCMV3-HA-hnRNPK S216(A/D) plasmids. Blots were developed using anti-hnRNPK, anti-PARP-1, or mouse anti-GAPDH as primary antibodies and Goat anti-mouse IgG-horseradish peroxidase (HRP) conjugate as the secondary antibodies.

**
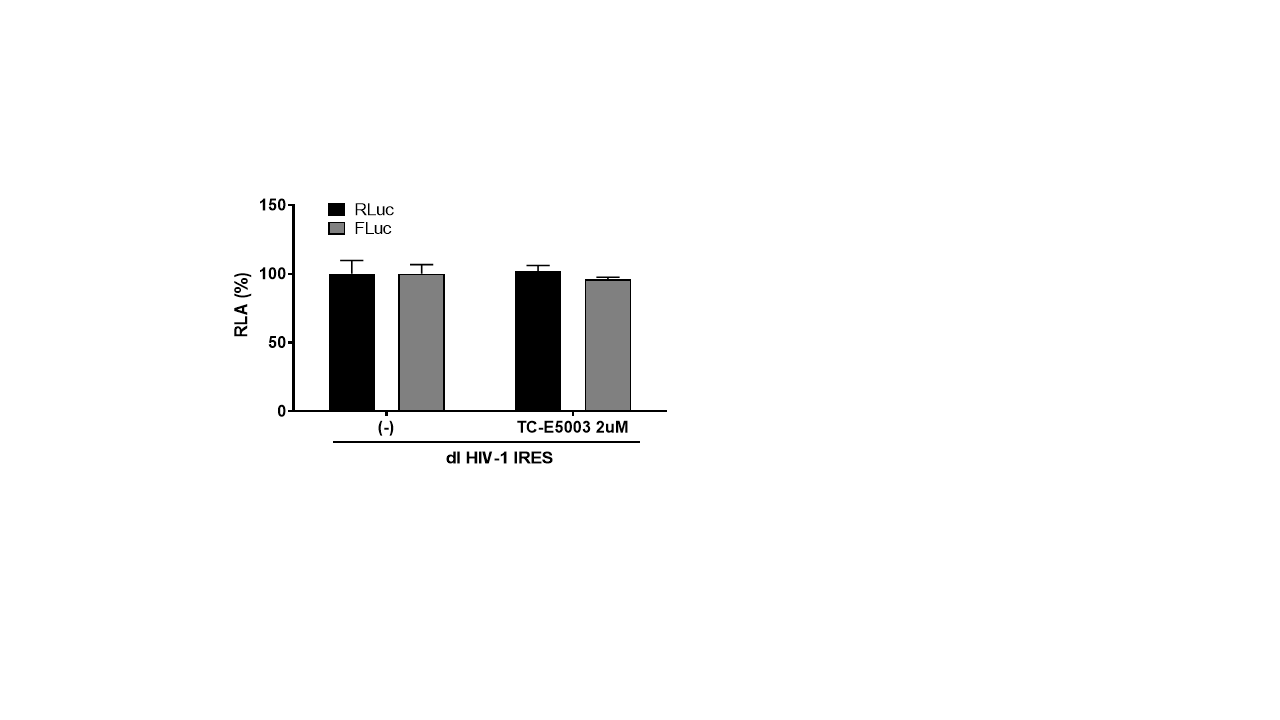
**

**Figure S4: TC-E-5003 does not inhibit RLuc or FLuc enzymatic activity.** HEK293T **c**ells were transfected with the dl HIV-1 IRES (200 ng) plasmid. TC-E-5003 (2 µM) was directly added, or not (-), to cell extracts recovered 24 hpt. RLuc and FLuc activities were measured 30 min post-incubation with the drug and expressed as RLA relative to values obtained when only the vehicle (-) was used, set to 100%. Statistical analysis was performed by a t-student test. Values shown are the mean (+/- SEM) from three independent experiments, each performed in duplicate.

**
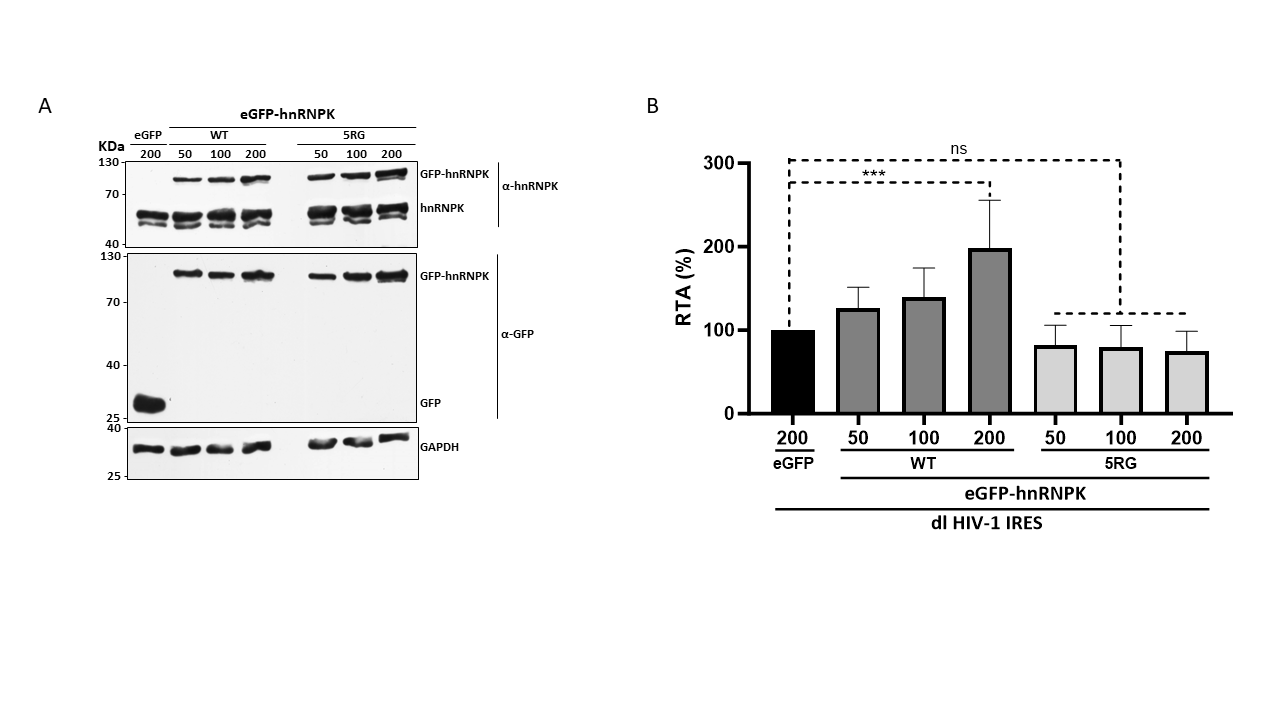
**

**Figure S5: HnRNPK 5RG does not stimulate dl HIV-1 IRES activity.** HEK293T cells were cotransfected with the dl HIV-1 plasmid (200 ng) and eGFP (200 ng) or increasing amounts (50, 100, 200 ng) of eGFP-hnRNPK (wt or 5RG) plasmid. Total protein extracts were prepared 24 hpt. **(A)** Western blot analysis was performed to detect the expression of total GFP, hnRNPK, and GFP-hnRNPK proteins using GAPDH as a loading control. **(B)** The RTA value in the absence of eGFP-hnRNPK was set to 100%. Values shown are the mean (+/- SEM) from eight independent experiments, with each performed in duplicate. Statistical analysis was performed by ordinary one-way ANOVA test (*** *P*≤0.001).

**
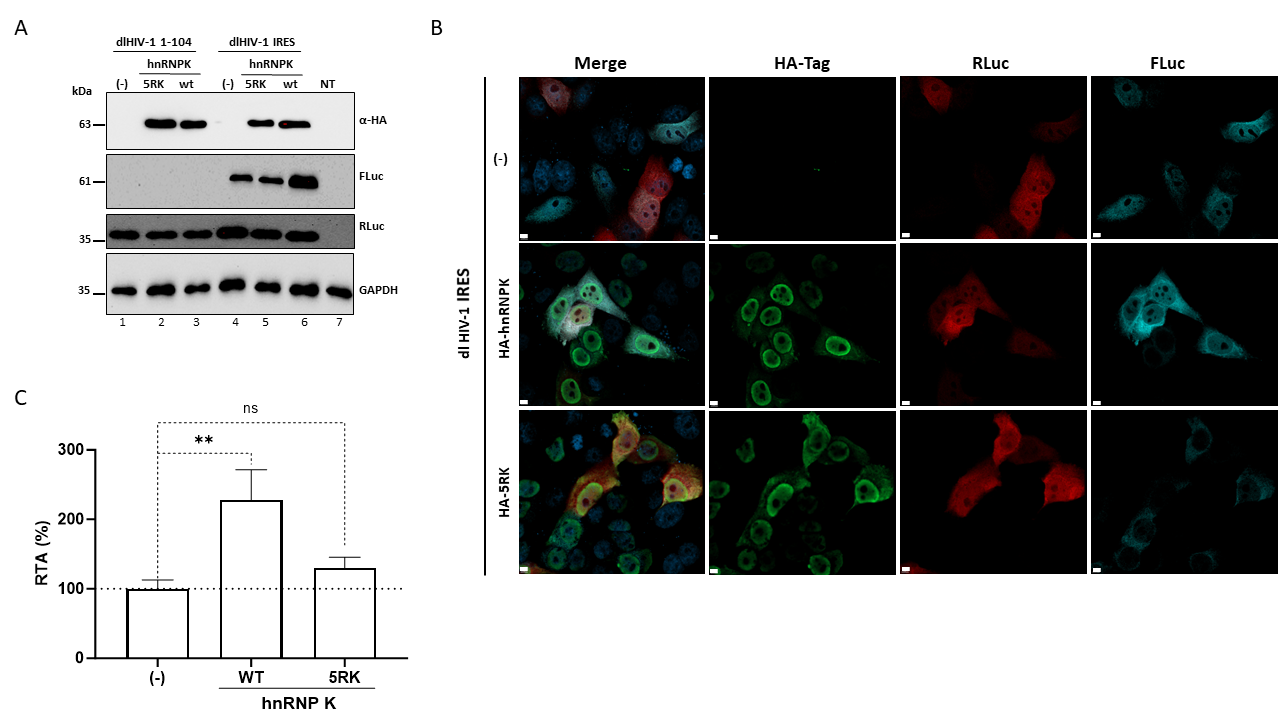
**

**Figure S6: HnRNPK, not the 5RK mutant, promotes HIV-1 IRES activity in HeLa cells.** HeLa cells were transfected with the dl HIV-1 1-104 or dl HIV-1 IRES plasmids, alone or with plasmids encoding for the HA-hnRNPK WT or the 5RK mutant. **(A)** Total protein extracts were prepared 24 hrs post-transfection and analyzed by Western blot to evaluate the expression of HA-recombinant proteins, FLuc and RLuc proteins, using GAPDH as a loading control. Extracts prepared from untransfected cells (-) were used as controls. **(B)** Immunofluorescence imaging; expression and localization of HA-hnRNPK or HA-5RK, RLuc, and FLuc. Size bars represent 5 µm. **(C)** From panel B, RTA was calculated by determining the ratio between FLuc and RLuc mean fluorescence intensity (MFI) divided by the individual cell's area (μm^2^). Statistical analysis was performed using a one-way ANOVA with Tukey post-test for multiple comparisons. ** = P ≤ 0.01, ns, nonsignificant.

**
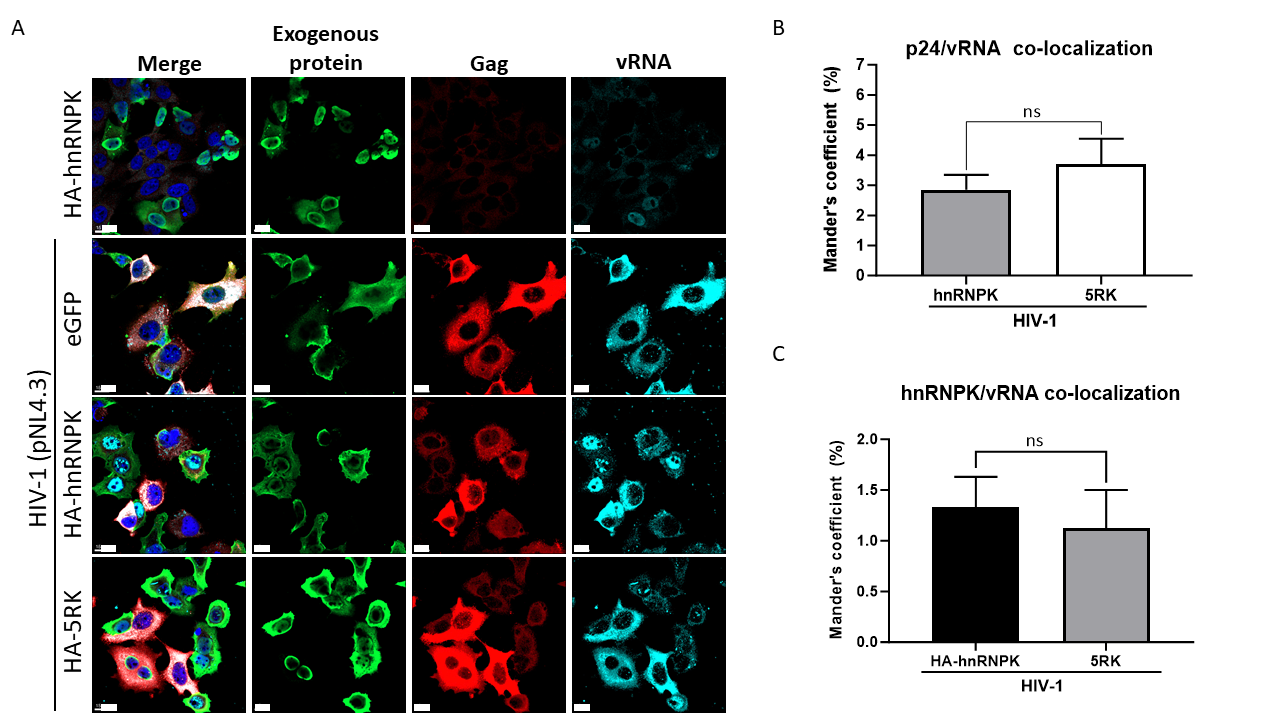
**

**Figure S7: HnRNPK and the 5RK mutant equivalently localize with the HIV-1 vRNA.** HeLa cells were cotransfected with the plasmid expressing the HA-hnRPK or HA-5RK proteins and the HIV-1-coding plasmid pNL4.3. A plasmid encoding for eGPF was used as a negative control (irrelevant protein). **(A)** The expression of HA-hnRNPK and HA-5RK proteins was visualized by IF. Viral replication was monitored by detecting HIV-1 Gag using an anti-p24 antibody and the vRNA by Fluorescence in situ hybridization (*FISH*). **(B-C)** Manders' coefficient, indicating the percentage of the overlapping signal of **(B)** HIV-1 Gag over the vRNA or **(C)** HA-hnRNPK WT or -5RK over HIV-1 vRNA, per frame. Size bars represent 15 µm. Statistical analysis was performed students' T-test with Welch's correction.

**
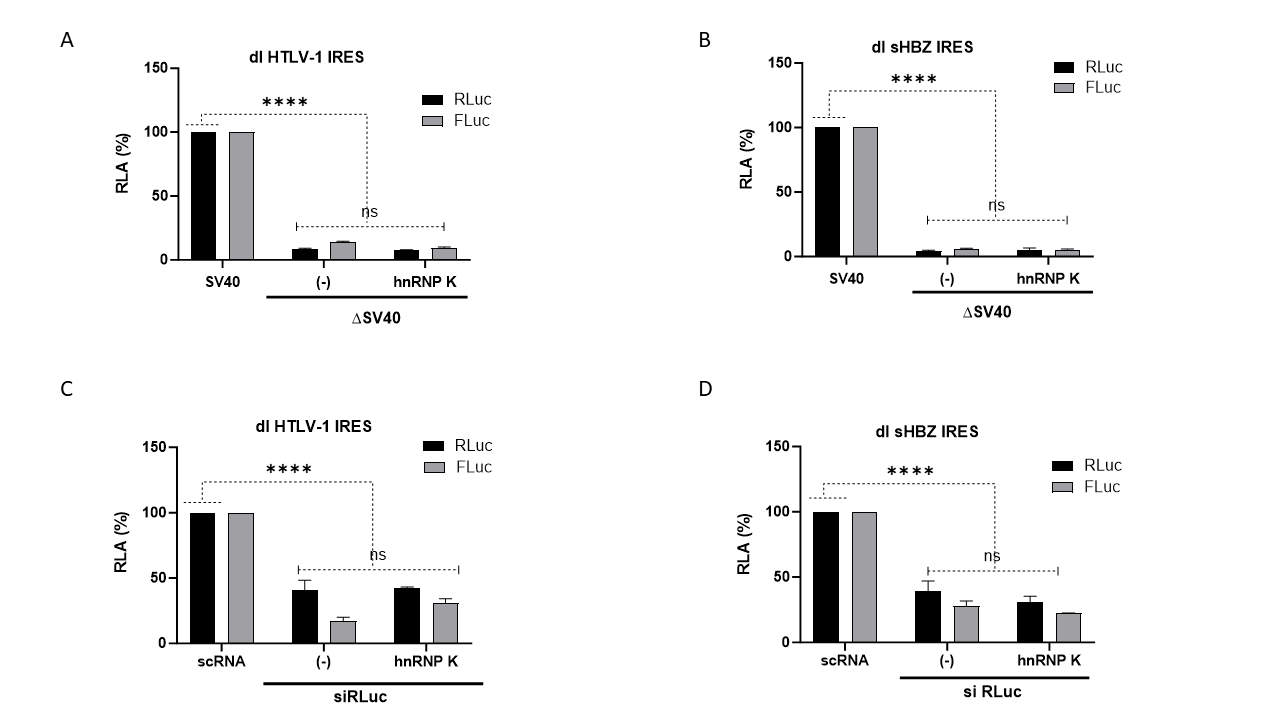
**

**Figure S8. Overexpression of hnRNPK does not enhance HTLV-1 IRES and dl sHBZ IRES DNA cryptic promoter activity or induce alternative splicing of their mRNAs. (A-B)**  HEK 293T cells were transfected with either the dl HTLV-1 IRES, dl sHBZ IRES, ∆SV40-dl HTLV-1 IRES, or ∆SV40-dl sHBZ IRES vector (150 ng) in the presence or the absence (-) of the HA-hnRNPK (100 ng) plasmid. 24 hpt total protein extracts were prepared. RLuc and FLuc activities were measured, and results are expressed as RLA relative to the activities obtained from the dl HTLV-1 IRES **(A)** or dl sHBZ IRES **(B)** vector when in the absence of the HA-hnRNPK, set to 100% (lower panel). Values shown are the mean (+/- SEM) from three independent experiments, with each performed in duplicate. Statistical analysis was performed by an ordinary two-way ANOVA test (**** = P<0.0001; ns, nonsignificant). **(C-D)** The dl HTLV-1 IRES **(C)** or dl sHBZ IRES (200 ng) **(D)** was cotransfected with a control scRNA (50 nM) or with siRLuc (50 nM), in the presence or the absence (-), of the HA-hnRNPK (200 ng) plasmid. 48 hpt, RLuc and FLuc activities were measured and expressed relative to the values obtained with scRNA, set to 100% (RLA) (lower panel). Values shown are the mean (+/- SEM) from three independent experiments, with each performed in duplicate. Statistical analysis was performed by an ordinary one-way ANOVA test (* = P<0.05; ns, nonsignificant).

**Supplemental Material and Methods: "H**eterogeneous nuclear ribonucleoprotein K **promotes cap-independent translation initiation of retroviral mRNAs." Fuentes, Olguín, et al. 2023.**

**Viral RNA purification and labeling**

For HIV-1 production and isolation of its genomic RNA, 1 million HEK293T cells were plated in 10-cm dishes and transfected with 10 µg of the HIV-1 coding plasmid pNL4-3 the following day. The supernatant was collected 48 hrs later and floating cells were removed by centrifugation at 3,000 rpm for 20 min, followed by filtration using a 0.45 mm filter. Then, viral particles present in the supernatant were concentrated by ultracentrifugation at 28,000 rpm for 1.5 h and resuspended in 1X D-PBS. Then, viral RNA was extracted by mixing 200 µL of concentrated virus with 1 mL Trizol (Invitrogen #15596026) and 200 µL of chloroform, centrifuged at 12,000 rpm for 15 min, and the aqueous phase was recovered and incubated with 500 mL isopropanol for 10 min at room temperature, followed by a last centrifugation at 12,000 rpm for 10 min at 4°C. The RNA pellet was washed using 1 mL of RNase-free 75% alcohol, centrifuged at 7,500 rpm for 5 min at 4°C, and resuspended in 50 µL RNase-free H_2_O. Viral RNA concentration was determined using a NanoDrop ND-100 spectrophotometer (ThermoFisher Scientific) and run on a 1% agarose gel to evaluate its integrity. For immunofluorescence purposes, the isolated viral RNA was subjected to an *in vitro* transcription step using the T7 RNA polymerase (ThermoFisher Scientific #EP011SKB008) in the presence of digoxigenin-labeled UTP (Roche # 3359247910). The labeled viral RNA was aliquoted, stored at -20°C, and used as a hybridization probe during FISH experiments.

**FISH Immunostaining and microscopy – hnRNPK Overexpression**

HeLa cells were seeded at 1x10^5^ cells/mL on 12-well plates (Sarstedt #83.3921) previously prepared with sterilized cover glasses of 0.15 mm thickness (VWR VistaVision #16004-300). The following day, cells were cotransfected with the HIV-1 enconding plasmid pNL4.3, or the empty vector pcDNA3.1 (0.5 µg per well), plus the plasmids coding for the HA-hnRNPK WT or HA-5RK mutant (0.2 µg per well). Transfections were carried out using JetPRIME® (Polyplus #101000027) according to the manufacturer's recommendations. 48- hours post-transfection, coverslips were washed twice with 1X D-PBS (multicell #311-425-CL) and fixed in 4% PFA for 15 mins, followed by 0.1M glycine for 10 mins and then 0.2% Triton-X for 5 mins, repeating the washing step in between reagents. All incubations were performed at room temperature. To detect HIV-1 RNA during staining, each coverslip was initially incubated with 25 units of DNAse I (Invitrogen #18047019) for 15 mins at room temperature, washed with 1X D-PBS and then incubated with the beforementioned synthetized HIV-1 viral RNA probe mixed in a hybridization solution (50% formamide, 1 µg/µL of tRNA, 5 units of RNAseOut, 5X Denhart's solution, and 25 ng of probe per cover glass) overnight at 42°C. The following day, coverslips were washed and blocked for 45 mins at room temperature in a 1% BSA solution (Thermo Scientific #37525) diluted in 1X D-PBS. After blocking, cells on cover glasses were incubated with a cocktail of primary antibodies (Rabbit anti-p24 (NIH HIV reagent program #ARP-4250), Mouse anti-HA (Sigma #H9658), and Sheep anti-Digoxigenin (Roche #57696520), all used at 1/200 dilution in blocking solution) for 1 hr at 37°C. Cover glasses were washed four times in 1X D-PBS for 5 mins and then incubated with a cocktail of secondary antibodies (Invitrogen Donkey anti-Mouse-AlexaFluor 488 [A-21202], Donkey anti-rabbit-AlexaFluor 594 [A-21207], and Donkey anti-sheep-AlexaFluor 647 [A-21448] all used at 1/400 dilution in blocking solution). The washing protocol was repeated, and cover glasses were incubated with DAPI (4',6-Diamidino-2-Phenylindole, Dihydrochloride) (Invitrogen #D1306), for 10 mins. Cover glasses were dried at room temperature, mounted using a drop of Immu-Mount (Thermo scientific #9990402), and sealed at the edges with regular nail polish. Images were acquired in the Zeiss LSM800 confocal microscope using immersion oil and a 40X/1.4 N.A objective.

For experiments using cells transfected with the HIV-1 IRES constructs, the same protocol beforementioned was followed, but the primary antibody cocktail was Rabbit anti-Renilla (abcam #ab185926), Mouse anti-Firefly (Millipore #L2164), Goat anti-HA (GeneScript #A00168), diluting the first two 1/100, and the last one 1/200, both in blocking solution.

**Cells segmentation and analysis**

To evaluate nuclear and cytoplasmic signal distribution, confocal images acquired from cotransfected HeLa cell's structure were segmented and analyzed using Imaris v10.0.1. Before cell segmentation, all pictures were analyzed for colocalizing signals between HIV-1 vRNA and p24, and HIV-1 vRNA and hnRNPK WT or mutant 5RK, setting threshold colocalizing signals based on nontransfected cells, keeping threshold values consistent between pictures from the same experiment. Then, "Cells" function was used to build an algorithm and separate cells cytoplasm and nucleus. To detect the nuclei, the DAPI channel was used as a reference, setting the nucleus diameter at 10 µm. Cell cytoplasm detection was carried out by using "Cell Body detection" and hnRNPK-HA signal as a source channel, and touching cells were split by seed points of 15 µm diameter. Colocalization between hnRNPK and vRNA was quantified by calculating Mander's coefficient per frame and expressed as a percentage.

In experiments where cells were transfected with the dl HIV-1 IRES constructs plus the HA-hnRNPK or HA-5RK expressing plasmids, the Relative Luciferase activity (RLA) was calculated by their fluorescent signal as follows. Using Imaris v10.0.1, "Surface" function was used to select Renilla and Firefly luciferases expressing cells and their respective MFI were subtracted per cell. A threshold fluorescent signal was established based on the Renilla signal, and data was normalized by dividing the signal per cell area (μm^2^). The RTA is calculated by the ratio of Fluc/Rluc signals per cell and represented as a Relative percentage to the control (HIV-1 IRES transfection only). Considering the co-transfection with HA-hnRNPK, HA-5RK, or GFP-expressing vectors, cells not expressing both constructs were excluded from the analysis.

**Western Blotting**

To evaluate intracellular levels of protein expression, HeLa were lysated by using NP40 lysis buffer supplemented with protease inhibitor and incubating the samples on ice for 30 mins, mixing them by vortex every 10 mins. Then, samples were centrifugated at 10,000g for 10 mins at 4°C to remove cell debris, and total amount of protein was quantified by Bradford reagent. 20 µg of protein were diluted in lab-made Laemmli buffer and distilled water until reaching a protein final concentration of 1 µg/µL. Diluted samples were boiled at 95°C for 5 mins and loaded into a 10 or 12% SDS-PAGE to further perform the electrophoresis at 100V. Later, in-gel samples were transferred to a nitrocellulose membrane by electrophoretic transfer at 250 mA for 3 hrs at 4°C. Protein transfer was confirmed by red Ponceau staining and further blocked with 5% Skim Milk prepared in TBS-T buffer (0.1% Tween 20) for 45 mins at room temperature. Blocked membranes were incubated with primary and secondary antibodies, being washed between antibodies with 1X TBS-T for 5 mins at room temperature. All antibodies were incubated for 2 hrs at room temperature or overnight at 4°C. Primary antibodies Mouse anti-24, Rabbit anti-Renilla, Mouse anti-Firefly and Rabbit anti-HA were all diluted in a solution of 1X TBS-T, 0.5% BSA and 3mM sodium azide 1/1000 times. Secondary antibodies, Donkey αRabbit-Ig and αMouse-Ig coupled to horseradish peroxidase, were all diluted 1/5000 in blocking solution. The signal was obtained by incubating the membranes with Western Lightning Pro, chemiluminescent Substrate, and captured using a ChemiDoc Imaging system (Bio-Rad) using the Chemiluminescence function, selecting "optimal Autoexposure" and 2x2 pixel binning settings.
